# Burden of esophageal cancer and its attributable risk factors in 204 countries and territories from 1990 to 2019

**DOI:** 10.3389/fpubh.2022.952087

**Published:** 2022-09-06

**Authors:** Yanqing Cai, Jianxiong Lin, Wenbo Wei, Peixing Chen, Kaitao Yao

**Affiliations:** ^1^Department of Medical Oncology, Jieyang People's Hospital, Jieyang, China; ^2^Department of Medical Oncology, The Second Affiliated Hospital of Shantou University Medical College, Shantou, China; ^3^Department of General Surgery, Jieyang People's Hospital, Jieyang, China

**Keywords:** esophageal cancer, Global Burden of Disease, trends, incidence, death, risk factor

## Abstract

**Background:**

Esophageal cancer is a global health concern. Regularly updated data about the burden of esophageal cancer are essential for formulating specific public policies. We aimed to estimate the global, regional, and national burden and trends of esophageal cancer and its attributable risk factors from 1990 to 2019, by age, sex and socio-demographic index (SDI).

**Methods:**

Data about the incidence, death, disability-adjusted life-years (DALYs), and age-standardized rates were collected from Global Burden of Disease study 2019. Estimated annual percentage changes were used to quantify the temporal trends of age-standardized rates. Moreover, the risk factors attributable to esophageal cancer deaths were also presented.

**Results:**

There were 534,563 incident cases and 498,067 deaths in 2019, contributing to 11,666,017 DALYs. The absolute numbers of incidence, death, and DALYs had increased from 1990 to 2019, contrasting with declined changes in their corresponding age-standardized rates. The burden of esophageal cancer varied across different regions and countries, and the age-standardized rates were negative with SDI. Almost half of the esophageal cancer was concentrated in China. Males accounted for most of the burden of esophageal cancer, and the onset age tended to be older. The death of esophageal cancer was primarily attributable to smoking, followed by alcohol use, high body mass index, diet low in fruits and diet low in vegetables.

**Conclusion:**

The burden of esophageal cancer was heterogeneous across regions and countries by sex, age, and SDI, providing information for governments that may help to formulate more targeted policies.

## Introduction

Esophageal cancer is a considerable contributor to the global cancer burden, with poor prognosis. According to GLOBOCAN 2020 (1), esophageal cancer has the seventh highest incidence of all cancers and is the sixth common cause of death. Since the clinical symptoms of early esophageal cancer are not obvious and specific, most patients are usually diagnosed at an advanced stage (2). Although early detection and treatment strategies have improved in recent years, the 5-year survival rate of esophageal cancer remains poor (2). The epidemiology of esophageal cancer presents marked geographical variability worldwide (1–4). Esophageal squamous cell carcinoma (ESCC) is the most common type in the world, while esophageal adenocarcinoma (EAC) is the dominant histological type in western countries. Recently, the incidence of EAC tends to grow in some western countries (2). Due to the increase in life expectancy and development of economy, significant changes have taken place in the epidemiology of esophageal cancer (1–4).

Although a recent study reported recent advances in the epidemiology of esophageal cancer using GLOBOCAN 2020 data, the comparing at national level is flawed, because the rates were not age-standardized (1). Thus far, two studies have used the Global Burden of Disease (GBD) 2017 data to show the burden of esophageal cancer (3, 5). However, some new data sources were added and methodological improvements applied in GBD 2019 (6, 7). More specifically, we aimed to provide the most up-to-date estimates incidence, death, and disability-adjusted life-years (DALYs) for esophageal cancer at global, regional and national levels in terms of counts and age-standardized rates between 1990 and 2019 by age, sex and sociodemographic index (SDI). Moreover, we also analyzed the contributions of risk factors to the deaths of esophageal cancer among different SDI regions.

## Materials and methods

### Data sources

Incidence, death, DALYs, and their age-standardized rates (ASRs) of esophageal cancer from 1990 to 2019 were obtained from the GBD study 2019 (http://ghdx.healthdata.org/gbd-results-tool) (6). Information about the sex, age, and risk factors were also acquired to estimate the burden of esophageal cancer. The GBD 2019 study assessed the burden of esophageal cancer from 204 countries and territories. In the framework of GBD, the world was divided into seven super-regions and 21 GBD regions based on geography. Meanwhile, the countries were divided into five quintiles according to SDI: high SDI, high-middle SDI, middle SDI, low-middle SDI and low SDI countries. SDI, ranged from 0 to 1, is a summary measure of a country's degree of development according to its geometric average of total fertility, per capita income, and mean years of education.

### Estimation framework

The details about the methodology of GBD study have been described in previous studies (6–8). Briefly, the data for mortality was extracted from vital registration, verbal autopsy, and cancer registry. Those sources that had both incidence and mortality on the same year would be used to calculate mortality-to-incidence ratios (MIRs). MIRs were initially modeled using a linear-step mixed-effects model with logit link functions, with Healthcare Access and Quality, age and sex as covariates. The estimates from this model would be smoothed over time and space, and adjusted by spatiotemporal Gaussian process regression. Both observed and estimated mortality (computed from MIRs and incidence data) were entered into Cause of Death Ensemble model. After adjusted by the CoDCorrect algorithm, the sum of all single causes was the estimated all-cause mortality. Esophageal cancer incidence was obtained by dividing the final mortality by the estimated MIRs. Prevalence was divided as four different health states, including diagnosis or treatment, remission, metastatic, and terminal phase. Years lived with disability (YLDs) were calculated by multiplying these health states with their disability weights, and years of life lost (YLLs) were estimated by multiplying the estimated number of deaths by standard life expectancy for corresponding age. Then, DALYs were the sum of YLDs and YLLs.

The proportion of esophageal cancer attributable to 84 environmental, occupational, metabolic, and behavioral risk factors were quantified using a comparative risk assessment approach (7). Population attributable fractions were concluded from systematic literature review, and a national mapping was accounted. The percentage of death of esophageal cancer attributed to the following five risk factors was reported: smoking, alcohol use, high body mass index (BMI), diet low in fruits and diet low in vegetables. Detailed descriptions about the estimation process are presented in the Supplementary Text S1.

### Statistical analysis

Incidence, death, DALYs, and their ASRs were the main index to evaluate the burden of esophageal cancer. All estimates were reported with 95% uncertainty interval (UI). The 95% UI was defined by the 2.5 and 97.5 centile values of 1,000 draw-level estimates after ordering from smallest to largest. A 95% UI, excluding 0, was considered as to be statistically significant. Moreover, estimated annual percentage changes (EAPC) tracked the dynamic changes of ASRs within a specified time interval (9). EAPC was estimated using a linear regression model: y = α + βx + ε, and y refers to ln (ASR), x represents calendar year, while ε represents error term. Then, EAPC was calculated as 100^*^(10^∧^β-1), and 95% confidence interval (CI) could be obtained from the model. If EAPC and its lower 95% CI were positive, ASRs were identified as on an upward trend. Contrarily, if EAPC and its upper 95% CI were negative, ASRs were identified as on a descending trend. Finally, we calculated the Pearson correlation coefficient between ASRs and SDI value to examine the correlation between ASRs and the development of society. All statistical analyses were performed by R program (Version 3.6.1). The *p*-value< 0.05 was recognized as statistically significant.

## Results

### Global burden of esophageal cancer

In 2019, there were 534,563 (95% UI 466,513–595,342) incident cases of esophageal cancer and 498,067 (95% UI 438,411–551,462) deaths globally, contributing to 11,666,017 (95% UI 10,378,747–12,938,949) DALYs (Table 1). Global incident cases increased by 67.07% (95% UI 46.51–98.53%) from 1990 to 2019, while age-standardized incidence rate (ASIR) decreased to 6.51 (95% UI 5.69–7.25) per 100,000 persons in 2019, with EAPC of −0.91 (95% CI −1.19 to −0.61) (Tables 1, 2). The death increased by 55.97% (95% UI 36.60–88.22%) between 1990 and 2019, with the age-standardized death rate (ASDR) decreasing to 6.11 (95% UI 5.38–6.76) per 100,000 persons in 2019 (EAPC: −1.18; 95% CI −1.48 to −0.89) (Tables 1, 2). DALYs of esophageal cancer increased by 42.13% (95% UI 23.09–75.52%) (Table 2), with a steeper increase in YLDs than in YLLs (76.37 vs. 41.77% increase). The age-standardized DALY rate decreased during study period, with EAPC of −1.41 (95% CI −1.72 to −1.11) (Tables 1, 2).

**Table 1:** Esophageal cancer incidence cases, age-standardized incidence rate, deaths, age-standardized death rate, disability-adjusted life-years, and age-standardized disability-adjusted life-years rate in 2019.

**Characteristics**	**Incidence cases** **(95%UI)**	**ASIR per 10^5^** **(95% UI)**	**Deaths** **(95% UI)**	**ASDR per 10^5^** **(95% UI)**	**DALY** **(95% UI)**	**Age-standardized DALY rate per 10^5^** **(95% UI)**
Global	534,563 (466,513–595,342)	6.51 (5.69–7.25)	498,067 (438,411–551,462)	6.11 (5.38–6.76)	11,666,017 (10,378,747–12,938,949)	139.79 (124.44–154.98)
**Sex**						
Male	388,827 (335,510–444,000)	10.13 (8.73–11.56)	365,554 (315,014–415,028)	9.68 (8.34–10.96)	8,821,716 (7,626,694–10,090,930)	221.38 (191.21–252.21)
Female	145,736 (119,952–165,068)	3.33 (2.74–3.77)	132,513 (110,337–150,271)	3.02 (2.52–3.43)	2,844,300 (2,434,410–3,195,247)	65.29 (55.83–73.33)
**SDI**						
High	95,911 (86,719–105,092)	5.20 (4.71–5.70)	79,088 (73,600–83,089)	4.18 (3.93–4.38)	1,653,972 (1,570,861–1,731,345)	95.82 (91.39–100.45)
High-middle	145,151 (113,067–169,189)	7.06 (5.50–8.22)	135,757 (108,339–156,606)	6.62 (5.29–7.62)	3,105,596 (2,487,365–3,596,286)	151.03 (121.17–174.68)
Middle	170,414 (142,732–194,526)	7.02 (5.81–7.99)	193,720 (157,830–223,774)	8.15 (6.54–9.39)	4,485,644 (3,737,169–5,192,821)	175.17 (145.44–202.51)
Low-middle	59,434 (52,249–83,651)	4.34 (3.82–6.14)	60,670 (53,987–85,565)	4.53 (4.02–6.39)	1,611,655 (1,433,392–2,250,321)	111.27 (99.04–155.69)
Low	25,861 (21,419–30,571)	4.96 (4.14–5.84)	28,684 (23,834–34,252)	5.69 (4.75–6.75)	805,543 (662,160–973,246)	141.09 (116.53–168.89)
**Region**						
Central Asia	4,834 (4,274–5,679)	6.70 (5.95–7.75)	4,924 (4,359–5,769)	7.08 (6.32–8.15)	129,818 (114,167–153,552)	164.79 (145.56–193.06)
High-income Asia Pacific	25,159 (21,213–29,616)	5.71 (4.83–6.76)	16,337 (14,650–17,795)	3.53 (3.22–3.84)	306,118 (281,921–333,763)	75.76 (70.65–82.63)
South Asia	53,488 (46,152–72,051)	3.78 (3.27–5.10)	54,161 (46,992–72,771)	3.93 (3.41–5.25)	1,476,590 (1,282,692–1,962,191)	98.29 (85.53–131.08)
East Asia	284,908 (220,166–338,886)	13.72 (10.64–16.25)	263,307 (209,014–314,860)	12.96 (10.19–15.37)	5,922,865 (4,733,467–7,156,234)	275.44 (221.92–331.75)
Southeast Asia	15,543 (13,193–18,202)	2.54 (2.18–2.97)	15,330 (13,164–17,964)	2.59 (2.24–3.05)	403,725 (342,843–472,284)	61.73 (52.74–72.38)
Oceania	147 (110–196)	2.15 (1.66–2.89)	147 (111–197)	2.30 (1.78–3.05)	4,213 (3,133–5,650)	53.58 (40.42–71.74)
Australasia	2,192 (1,767–2,707)	4.41 (3.55–5.46)	2,035 (1,830–2,218)	4.02 (3.65–4.39)	39,885 (36,426–43,385)	85.18 (78.02–92.42)
Central Europe	5,853 (5,109–6,664)	2.89 (2.52–3.30)	5,856 (5,124–6,670)	2.86 (2.49–3.25)	143,701 (124,717–164,319)	74.70 (64.49–85.35)
Eastern Europe	11,086 (9,669–12,604)	3.25 (2.83–3.69)	10,655 (9,298–12,077)	3.10 (2.71–3.51)	277,541 (240,922–316,272)	83.49 (72.58–95.11)
Western Europe	40,174 (35,133–45,706)	4.64 (4.06–5.29)	34,847 (32,416–36,620)	3.87 (3.64–4.06)	706,817 (669,630–741,655)	88.65 (84.34–92.92)
High-income North America	26,162 (22,461–30,594)	4.22 (3.63–4.96)	24,152 (22,876–25,147)	3.84 (3.65–3.99)	524,630 (503,915–544,030)	88.30 (84.99–91.37)
Caribbean	1,920 (1,641–2,200)	3.69 (3.15–4.23)	1,923 (1,649–2,197)	3.70 (3.17–4.23)	47,316 (40,107–54,718)	90.70 (76.86–104.79)
Andean Latin America	827 (670–1,021)	1.51 (1.22–1.85)	889 (723–1,090)	1.63 (1.33–2.00)	18,839 (15,081–23,638)	33.43 (26.85–41.96)
Central Latin America	3,869 (3,281–4,511)	1.66 (1.41–1.94)	4,021 (3,391–4,707)	1.74 (1.47–2.04)	90,775 (76,781–107,044)	37.96 (32.14–44.75)
Tropical Latin America	12,684 (11,993–13,294)	5.17 (4.87–5.42)	12,767 (11,996–13,448)	5.25 (4.92–5.53)	328,430 (311,722–345,187)	131.03 (124.29–137.70)
Southern Latin America	3,945 (3,158–4,943)	4.70 (3.75–5.89)	4,067 (3,769–4,359)	4.82 (4.47–5.15)	83,206 (77,617–89,098)	101.08 (94.41–108.18)
North Africa and Middle East	10,024 (7,415–11,436)	2.36 (1.79–2.66)	9,968 (7,385–11,383)	2.44 (1.85–2.75)	259,488 (183,343–301,673)	55.58 (40.51–63.78)
Central Sub-Saharan Africa	4,431 (2,378–6,020)	8.41 (4.48–11.59)	4,509 (2,426–6,141)	8.98 (4.79–12.41)	127,510 (68,514–172,764)	215.22 (115.53–294.00)
Eastern Sub-Saharan Africa	16,391 (12,431–20,713)	10.03 (7.71–12.60)	16,940 (12,941–21,344)	10.77 (8.28–13.48)	476,744 (361,802–608,685)	263.43 (200.49–332.94)
Southern Sub-Saharan Africa	5,941 (5,316–6,943)	10.66 (9.56–12.29)	6,095 (5,489–7,002)	11.30 (10.23–12.77)	159,882 (142,561–188,481)	267.11 (239.24–310.78)
Western Sub-Saharan Africa	4,986 (3,776–5,992)	2.71 (2.06–3.21)	5,135 (3,916–6,146)	2.89 (2.20–3.42)	137,923 (104,912–167,521)	67.83 (51.54–81.61)

ASIR, age-standardized incidence rate; ASDR, age-standardized deaths rate; DALY, disability-adjusted life-years; SDI, Socio-demographic index; UI, uncertainty interval.

**Table 2:** The trends in incidence, death and DALY of esophageal cancer between 1990 and 2019.

**Characteristics**	**Relative change in incidence** **(95% UI)**	**EAPC of ASIR** **(95% CI)**	**Relative change in death** **(95% UI)**	**EAPC of ASDR** **(95% CI)**	**Relative change in DALY** **(95% UI)**	**EAPC of age-standardized DALY rate** **(95% CI)**
Global	67.07% (46.51–98.53%)	−0.91 (−1.19 to −0.61)	55.97% (36.60–88.22%)	−1.18 (−1.48 to −0.89)	42.13% (23.09–75.52%)	−1.41 (−1.72 to −1.11)
**Sex**						
Male	79.97% (52.61–123.84%)	−0.60 (−0.86 to −0.32)	70.50% (44.17–108.99%)	−0.84 (−1.11 to −0.56)	53.81% (29.46–93.25%)	−1.08 (−1.37 to −1.08)
Female	40.25% (19.02–73.46%)	−1.66 (−2.00 to −1.31)	26.29% (8.40–57.13%)	−2.07 (−2.41 to −1.72)	15.03% (−1.97 to47.53%)	−2.31 (−2.65 to −2.31)
**SDI**						
High	83.89% (68.21–100.91%)	0 (−0.15 to 0.15)	66.72% (59.56–73.62%)	−0.44 (−0.53 to −0.35)	48.18% (42.36–54.76%)	−0.65 (−0.73 to −0.56)
High-middle	67.35% (41.21–99.15%)	−0.62 (−0.89 to −0.33)	54.07% (31.08–81.70%)	−0.98 (−1.26 to −0.69)	38.94% (16.75–64.81%)	−1.20 (−1.53 to −0.89)
Middle	25.08% (2.57–88.13%)	−2.17 (−2.63 to −1.7)	40.16% (14.98–107.41%)	−2.11 (−2.55 to −1.66)	25.49% (1.50–91.19%)	−2.40 (−2.86 to −1.95)
Low-middle	95.54% (67.51–126.15%)	−0.66 (−0.75 to −0.56)	96.71% (70.18–127.27%)	−0.70 (−0.79 to−0.60)	86.69% (60.52–116.79%)	−0.70 (−0.79 to −0.61)
Low	80.02% (55.00–110.79%)	−0.48 (−0.55 to −0.4)	95.90% (68.89–129.45%)	−0.49 (−0.55 to −0.43)	93.09% (64.13–131.33%)	−0.53 (−0.60 to −0.46)
**Region**						
Central Asia	−24.65% (−33.13 to −12.29%)	−2.85 (−3.09 to −2.6)	−25.63% (−33.80 to −13.66%)	−2.83 (−3.07 to −2.59)	−24.00% (−33.01 to −10.75%)	−2.99 (−3.24 to −2.76)
High-income Asia Pacific	90.63% (61.41–124.02%)	−0.57 (−0.75 to −0.39)	61.23% (48.13–76.23%)	−1.44 (−1.53 to −1.35)	25.53% (17.01–41.42%)	−1.77 (−1.91 to −1.65)
South Asia	108.90% (74.64–149.22%)	−0.86 (−0.99 to −0.72)	109.60% (74.75–148.32%)	−0.93 (−1.07 to −0.79)	98.34% (65.58–135.09%)	−0.84 (−0.95 to −0.71)
East Asia	61.66% (29.88–115.34%)	−1.54 (−2 to −1.07)	47.03% (18.02–101.56%)	−1.92 (−2.39 to −1.44)	29.80% (1.81–85.78%)	−2.21 (−2.71 to −1.70)
Southeast Asia	119.22% (81.89–163.71%)	−0.34 (−0.39 to −0.29)	113.47% (77.30–154.57%)	−0.45 (−0.50 to −0.40)	100.75% (64.83–141.44%)	−0.59 (−0.64 to −0.53)
Oceania	132.82% (88.76–191.04%)	−0.05 (−0.08 to −0.03)	132.70% (89.76–188.36%)	−0.06 (−0.08 to −0.04)	130.54% (84.02–189.65%)	−0.10 (−0.14 to −0.07)
Australasia	103.66% (65.49–151.84%)	−0.3 (−0.4 to −0.19)	100.33% (83.67–119.07%)	−0.41 (−0.48 to −0.33)	79.57% (65.27–95.53%)	−0.51 (−0.57 to −0.44)
Central Europe	36.84% (19.75–55.28%)	−0.17 (−0.27 to −0.06)	35.28% (18.64–52.93%)	−0.28 (−0.37 to −0.18)	24.54% (8.16–41.71%)	−0.37 (−0.49 to −0.24)
Eastern Europe	−8.87% (−19.31 to 2.56%)	−1.38 (−1.63 to −1.12)	−12.27% (−23.37 to −0.73%)	−1.58 (−1.82 to −1.34)	−13.51% (−24.70 to −1.54%)	−1.51 (−1.77 to −1.25)
Western Europe	48.82% (30.69–68.52%)	−0.23 (−0.35 to −0.12)	34.26% (28.07–40.07%)	−0.71 (−0.79 to −0.64)	17.55% (12.49–22.67%)	−0.98 (−1.06 to −0.89)
High-income North America	98.14% (69.72–131.37%)	0.19 (0.06–0.32)	92.98% (85.93–100.79%)	0.09 (−0.02 to 0.20)	79.23% (72.65–86.51%)	−0.14 (−0.24 to −0.05)
Caribbean	94.99% (66.61–126.58%)	0.07 (−0.14 to 0.28)	88.17% (61.98–117.70%)	−0.11 (−0.32 to 0.10)	94.76% (65.15–126.51%)	0.17 (−0.04 to 0.38)
Andean Latin America	114.37% (69.16–176.50%)	−0.85 (−0.94 to −0.76)	115.74% (72.17–175.94%)	−0.88 (−0.97 to −0.79)	90.49% (47.38–149.93%)	−1.12 (−1.23 to −1.02)
Central Latin America	102.65% (71.93–136.58%)	−1.48 (−1.6 to −1.36)	100.29% (70.55–133.73%)	−1.60 (−1.72 to −1.48)	88.16% (59.24–122.30%)	−1.54 (−1.66 to −1.41)
Tropical Latin America	106.88% (93.93–119.17%)	−0.85 (−0.89 to −0.79)	105.75% (93.20–119.03%)	−0.93 (−0.97 to −0.88)	93.10% (81.44–105.23%)	−0.94 (−1.00 to −0.87)
Southern Latin America	16.64% (−7.70 to 45.90%)	−1.93 (−2.1 to −1.76)	14.79% (6.72–23.36%)	−2.05 (−2.22 to −1.88)	3.44% (−4.04 to 10.92%)	−2.22 (−2.38 to −2.06)
North Africa and Middle East	129.34% (88.87–174.68%)	−0.32 (−0.36 to −0.27)	124.43% (85.92–167.94%)	−0.38 (−0.41 to −0.34)	112.06% (73.71–154.89%)	−0.66 (−0.71 to −0.60)
Central Sub-Saharan Africa	78.54% (34.04–142.28%)	−1.09 (−1.18 to −1)	78.36% (35.90–140.82%)	−1.08 (−1.18 to −1.00)	75.98% (31.41–144.00%)	−1.20 (−1.30 to −1.11)
Eastern Sub-Saharan Africa	94.25% (61.34–138.00%)	−0.46 (−0.54 to −0.38)	96.50% (64.89–138.60%)	−0.39 (−0.46 to −0.32)	94.62% (60.22–142.90%)	−0.49 (−0.57 to −0.40)
Southern Sub-Saharan Africa	59.56% (33.02–118.58%)	−1.35 (−1.92 to −0.79)	61.92% (34.42–118.29%)	−1.27 (−1.84 to −0.70)	49.46% (24.68–107.66%)	−1.61 (−2.18 to −1.02)
Western Sub-Saharan Africa	171.31% (100.28–228.89%)	1.16 (1.03–1.29)	170.25% (104.49–225.96%)	1.19 (1.05–1.31)	170.39% (106.07–230.71%)	1.04 (0.91–1.15)

ASIR, age-standardized incidence rate; ASDR, age-standardized deaths rate; CI, confidential interval; DALY, disability-adjusted life-years; EAPC, estimated annual percentage change; SDI, Socio-demographic index; UI, uncertainty interval.

The incident cases of esophageal cancer increased in all SDI countries. ASIR decreased the fastest in the middle SDI countries (EAPC = −2.17, 95% CI −2.63 to −1.7), and plateaued in high SDI region (EAPC = −0, 95% CI −0.15 to 0.15) (Table 2). The deaths and DALYs had increasing trend in all SDI countries, while ASDR and age-standardized DALY rate decreased in all SDI countries, especially in middle SDI countries (Table 2).

### Regional and national burden of esophageal cancer

**Figure 1:**
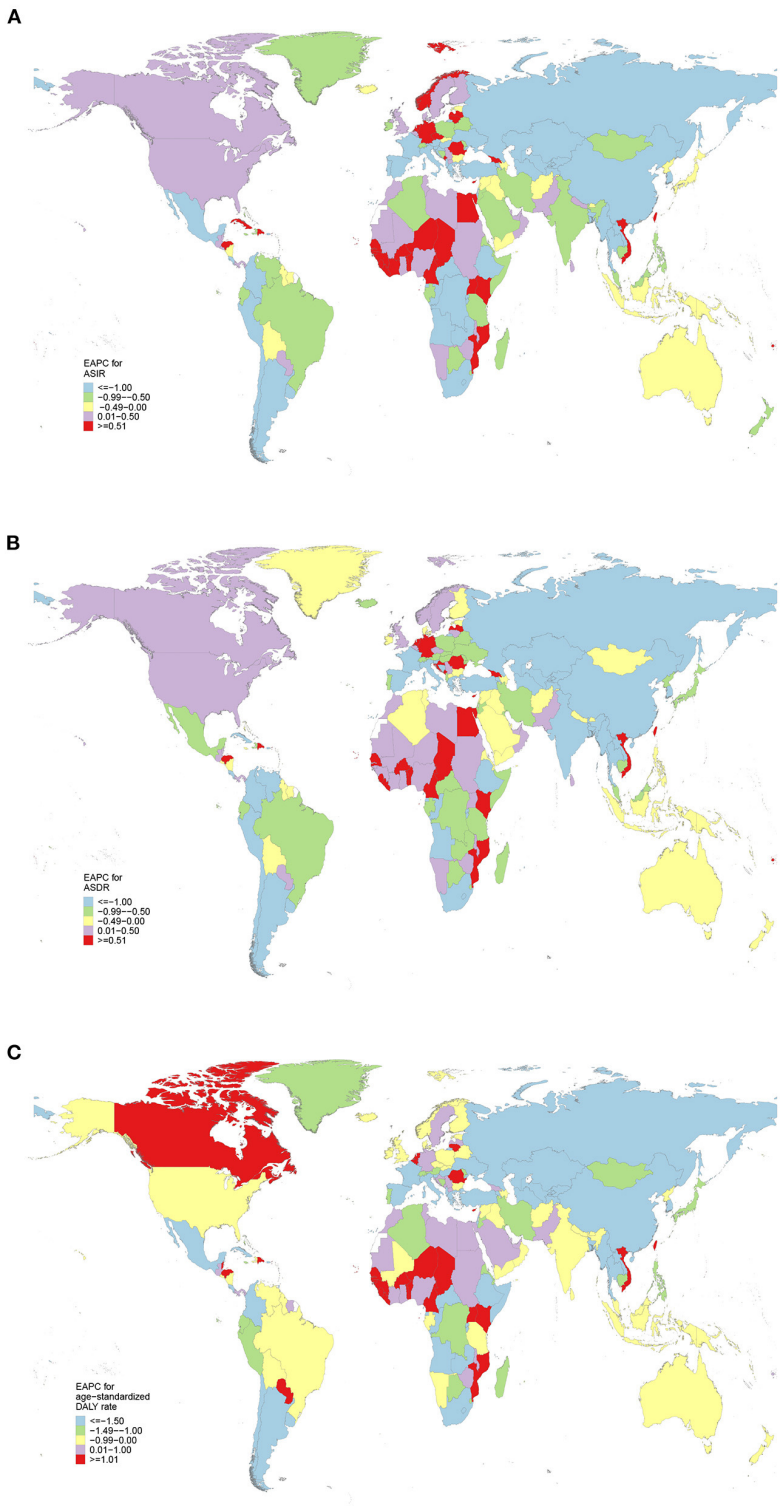
The estimated annual percentage changes (EAPCs) of esophageal cancer at national levels from 1990 to 2019. **(A)** The EAPC of age-standardized incidence rate (ASIR). **(B)** The EAPC of age-standardized death rate (ASDR). **(C)** The EAPC of age-standardized disability-adjusted life-year (DALY) rate.

The highest ASIR [13.72 (95% UI 10.64–16.25)] was estimated in East Asia (Table 1). Between 1990 and 2019, the incident cases showed a downward trend in Central Asia [−24.65% (95% UI −33.13 to −12.29%)] (Table 2). Decreasing trend of ASIR was detected in 18 regions, with the largest decrease in Central Asia (EAPC = −2.85, 95%CI −3.09 to −2.6) (Table 2). ASIR tended to be stable in Caribbean (EAPC = 0.07, 95%CI −0.14 to 0.28) (Table 2). More than half of the newly cancer cases were reported in China in 2019 [278,121 (95% UI 213,512–331,600)], followed by India and United States of America (Supplementary Table S1; Supplementary Figure S1). Among 204 countries/territories, ASIR in 2019 varied from 24.53 (95% UI 18.74–32.51) per 100,000 persons in Malawi to 0.91 (95% UI 0.65–1.58) per 100,000 persons in Nigeria (Supplementary Table S1; Supplementary Figure S1). Moreover, 119 countries experienced a significant decrease in the ASIR, with the highest in Turkmenistan (EAPC = −4.46, 95%CI −5.23 to −3.67) (Supplementary Table S2; Figure 1). In contrast, increasing trends were observed in 58 countries, particularly in Northern Mariana Islands (EAPC = 3.14, 95% CI 2.69–3.59) (Supplementary Table S2; Figure 1).

Similarly, East Asia had the highest ASDR [12.96 (95% UI 10.19–15.37)] in 2019 (Table 1). Only in Central Asia [−24.65% (95% UI −33.13 to −12.29%)] and Eastern Europe [−24.65% (95% UI −33.13 to −12.29%)], the death cases decreased between 1990 and 2019 (Table 2). The ASDR decreased in most areas, of which the most pronounced was in Central Asia (EAPC = −2.83, 95%CI −3.07 to −2.59) (Table 2). Increasing trend occurred in Western Sub-Saharan Africa (EAPC = 1.19, 95% CI: 1.05–1.31) (Table 2). China, India and United States of America, which had the largest population in the world, had the most deaths of esophageal cancer in 2019 (Supplementary Table S1; Supplementary Figure S2). Among 204 countries/territories, the highest ASDR in 2019 occurred in Malawi [25.76 (95% UI 19.76–33.94) per 100,000 persons] and the lowest in Syrian Arab Republic [0.96 (95% UI 0.73–1.23) per 100,000 persons] (Supplementary Table S1; Supplementary Figure 2). From 1990 to 2019, 123 and 53 countries had a significant decrease and increase in ASDR, respectively (Supplementary Table S2; Figure 1). Particularly, the highest increase and decrease was recorded in Northern Mariana Islands (EAPC = 3.10, 95% CI 2.64–3.55) and Turkmenistan (EAPC = −4.47, 95% CI −5.24 to −3.69), respectively (Supplementary Table S2).

East Asia also was the areas with the highest age-standardized DALY rate [275.44 (95% UI 221.92–331.75)] due to esophageal cancer in 2019 (Table 1). Decreasing trends of DLAYs were observed in Central Asia [−24.00% (95% UI −33.01 to −10.75%)] and Eastern Europe [−13.51% (95% UI −24.70 to −1.54%)] (Table 2). A notable decline was in the age-standardized DALY rate of Central Asia, with an EAPC of −2.99 (95% CI, −3.24 to −2.76) during the study period (Table 2). The highest DALYs were reported in China, India and United States of America in 2019 (Supplementary Table S1; Supplementary Figure S3). Besides, Malawi had the highest age-standardized DALY rate [651.57 (95% UI 481.63–882.94) per 100,000 persons], while Tunisia had the lowest age-standardized DALY rate [21.41 (95% UI 14.66–29.20) per 100,000 persons] in 2019 (Supplementary Table S1; Supplementary Figure S3). The EAPC of age-standardized DALY rate was highest in Northern Mariana Islands (EAPC = 3.04, 95% CI 2.58–3.5) (Supplementary Table S2; Figure 1).

### The correlation between SDI and the burden of esophageal cancer

**Figure 2:**
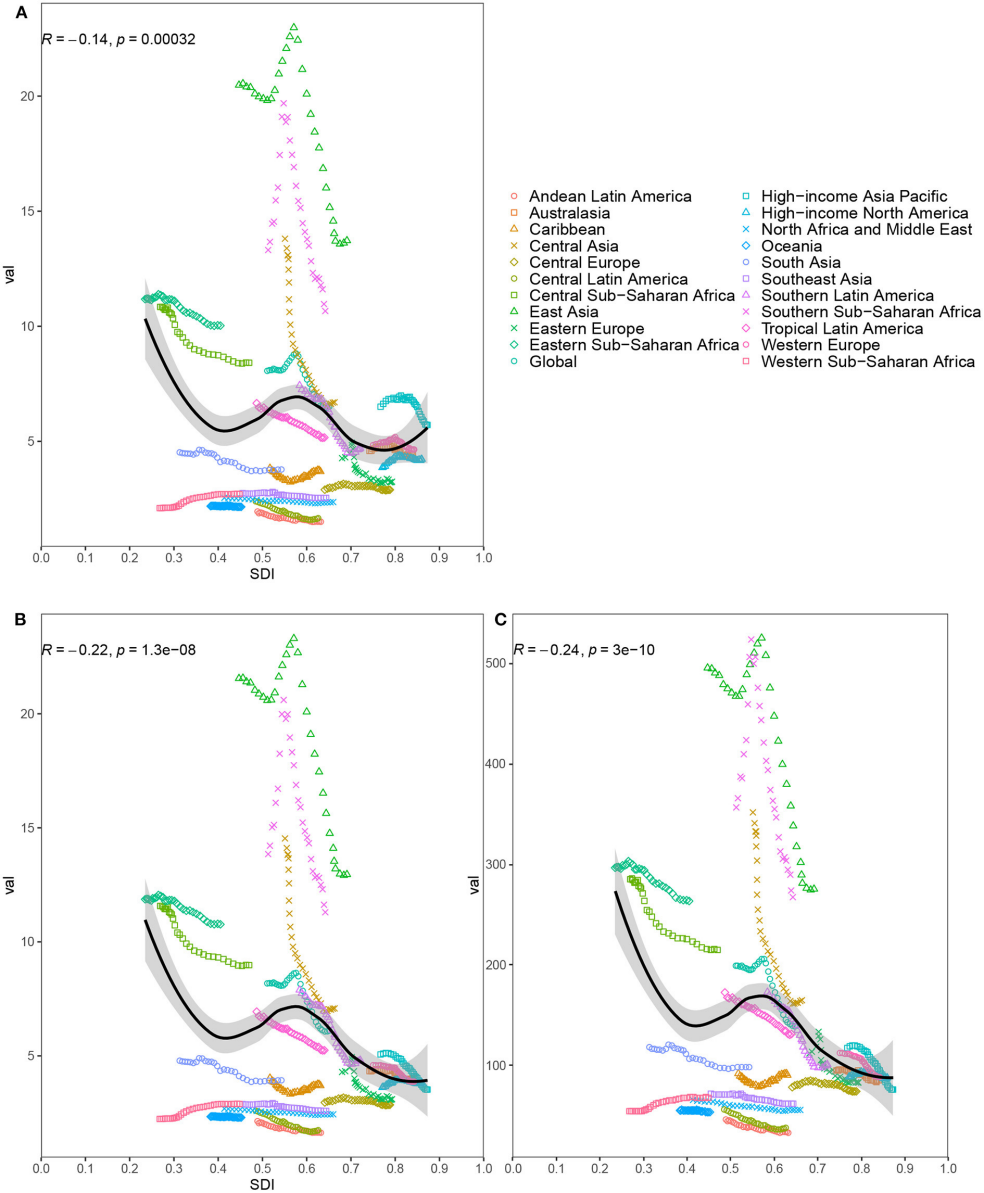
Age-standardized incidence rate **(A)**, age-standardized death rate **(B)** and age-standardized disability-adjusted life-year rate **(C)** for esophageal cancer for 21 regions by Socio-Demographic Index (SDI) from 1990 to 2019; The black line represents the expected values based on SDI and disease rates in all locations. Thirty points are plotted for each region and show observed rate from 1990 to 2019 for that region. The *R* indices and *P*-value were derived from Pearson correlation analysis.

We explored the relationship between SDI and ASRs in 21 GBD regions from 1990 to 2019 (Figure 2). The results found that the ASRs were negatively correlated with SDI. In particularly, East Asia and Southern Sub-Saharan Africa had much higher ASRs than expected values on the basis of SDI for nearly all years. The burden in East Asia and Southern Sub-Saharan Africa initially increased and then decreased with an increase in SDI over time. National-level analysis found that there was also a negative association between SDI and ASRs (Supplementary Figure S4). A part of countries had much higher ASRs than expected values based on SDI in 2019. And a high burden was not confined to developed or less developed countries.

### Age and sex patterns

**Figure 3:**
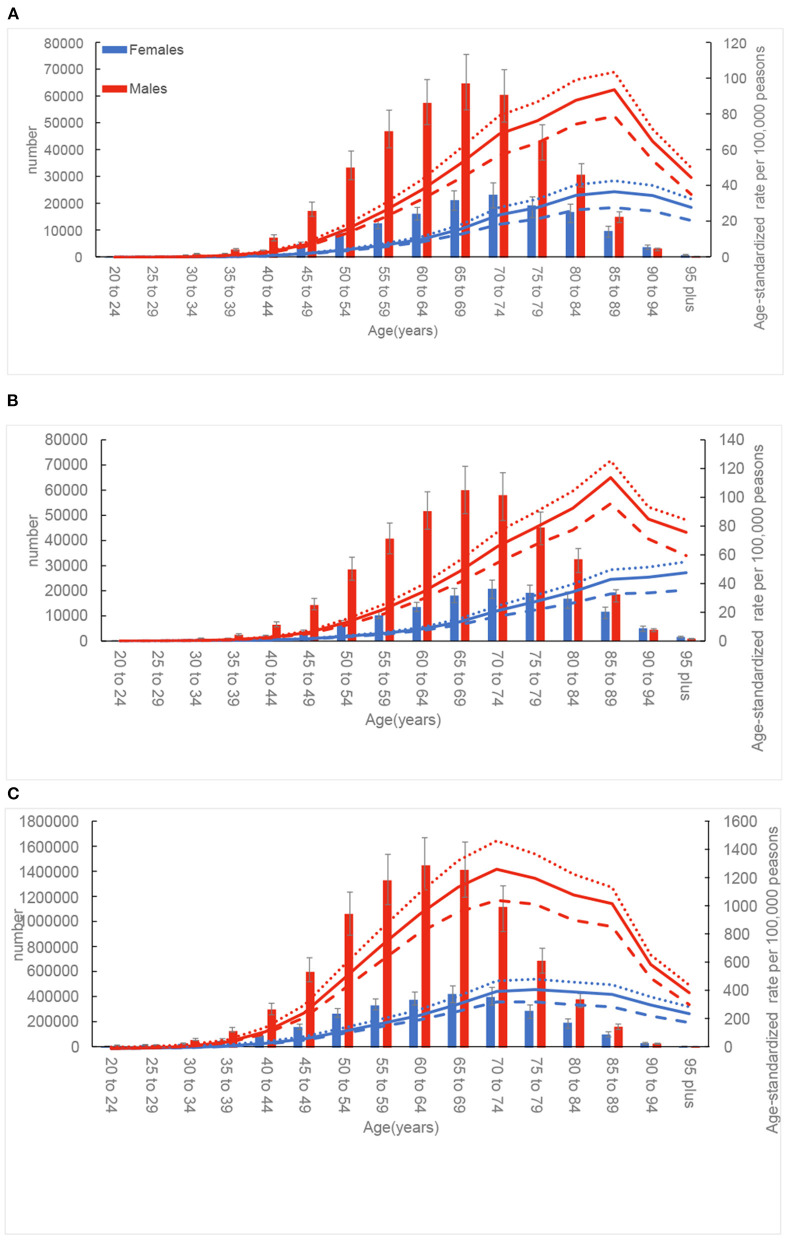
Global number and age-standardized rates of incidence **(A)**, death **(B)**, and disability-adjusted life-years (DALYs) **(C)** of esophageal cancer by age and sex in 2019; dotted and dashed lines indicate 95% upper and lower uncertainty intervals, respectively.

Globally, the ASRs were lower in females than in males across all age groups (Figure 3). The ASIR for both males and females increased with increasing age, peaking at the 85–89 age group; after this age, the trend declined. In addition, the difference in ASIR between females and males increased with each increasing age group up to the 85–89 age group, after which the gap began to decrease. A relatively similar pattern was also showed for ASDR in males. Unlike the ASIR, the ASDR for females increased in a non-linear manner with age. The age-standardized DALY rate reached the highest level at 65–69 age group for males and at 70–74 age group for females.

### Attributable risk factors

**Figure 4:**
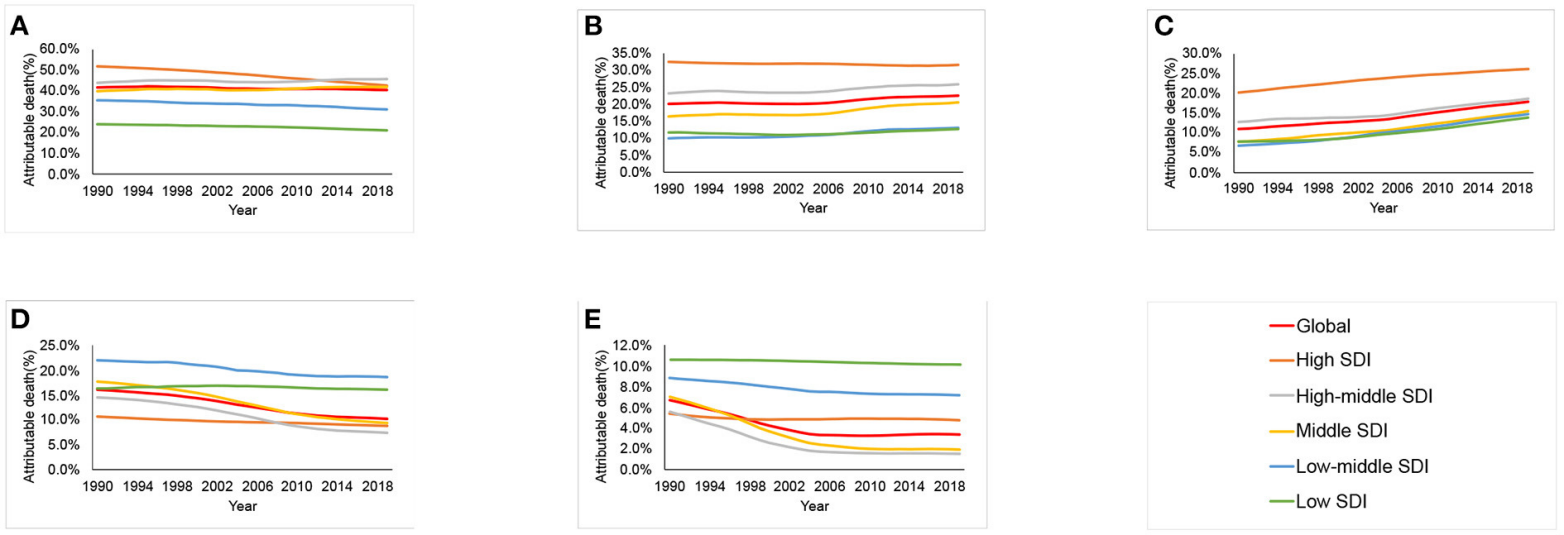
Contributions of different risk factors to esophageal cancer deaths globally and in five sociodemographic index (SDI) quintiles from 1990 to 2019. **(A)** Smoking. **(B)** Alcohol use. **(C)** High body mass index. **(D)** Diet low in fruits. **(E)** Diet low in vegetables.

Among all potentially modifiable risk factors quantified in GBD 2019, the deaths of esophageal cancer worldwide in 2019 were primarily attributable to smoking [40.6% (95% UI 36.8–44.3%)], followed by alcohol use [22.6% (95% UI 17.2–27.9%)], high BMI [17.9% (95% UI 5.7–35.0%)], diet low in fruits [10.3% (95% UI 3.1–22.2%)], and diet low in vegetables [3.5% (95% UI (0.5–6.9%)] (Supplementary Table S3). By SDI quintile, smoking, alcohol use and high BMI were the major risk factors in high, high-middle and middle SDI countries. The percentage contributions of diet low in vegetables and diet low in fruits to esophageal cancer related deaths were largest in low and low-middle SDI countries, respectively. Between 1990 and 2019, a downward trend was observed for smoking in high SDI countries (Figure 4). The deaths of esophageal cancer attributable to high BMI increased globally and in all SDI countries. The deaths attributable to alcohol increased in most countries, except for high SDI countries. The deaths of esophageal cancer attributable to diet low in vegetables and diet low in fruits decreased globally, but remained stable in low SDI countries.

## Discussion

Esophageal cancer remained a major global health problem, and the burden and trends varied across regions and countries. The burden of esophageal cancer had a negative association with the SDI. The ASIR, ASDR and age-standardized DALY rate were lower in females than males across all age groups, and the burden peaked in the elderly. Moreover, the deaths of esophageal cancer were primarily attributable to smoking, followed by alcohol use, high BMI, diet low in fruits and diet low in vegetables. However, the changes of the attributable burden to these risk factors during the study period were heterogeneous. Hence, timely information about the epidemiology of esophageal cancer is an appropriate approach to support the health planning and resource allocation.

Two similar studies have measured the burden of esophageal cancer utilizing data from the GBD study 2017 (3, 5). There still exist some differences of our study to previous studies. Firstly, we used the latest data from the GBD 2019, which improved the method and model of data correction compared with previous studies. And our study also covered more locations and data sources, including data from nine additional countries and territories which were newly added to the GBD 2019. Secondly, unlike the previous study (3), this study applied the EAPC to quantificationally describe the trend of incidence, death and DALY from 1990 to 2019. Thirdly, ASIR, ASDR, and age-standardized DALY rate and their correlation to SDI were all included in our study, while past study has only focused on the correlation of age-standardized DALY rate and SDI (3). Fourthly, the burden of esophageal cancer related to diet low in vegetables was firstly reported in our study. The burden of esophageal cancer related to diet low in vegetables was highest in low SDI countries. Lastly, we demonstrated that the trends of the different risk factors between 1990 and 2019 varied across different SDI countries.

The numbers of incidence, death, and DALYs had increased from 1990 to 2019, contrasting with declined changes in their ASRs. The exact causes for the disparities may be partly due to population growth and aging. An aging and growing population means that the numbers of incidence, death, and DALYs will continue to increase in many locations. Therefore, the decline in ASRs does not necessarily lead to a lower burden of esophageal cancer in high-risk country health systems.

Further investigation is needed to investigate the causes of the high ASRs found in some countries. Some countries in east and central Asia had high ASRs over the study period. Since at least the early 1970s, high rates of esophageal cancer have been noted in regions located along with so-called “Asian esophageal cancer belt” extending from China and Mongolia to the Caspian Sea (10, 11). In addition, esophageal cancer is prevalent in eastern, central, and southern Sub-Saharan Africa, known as the African esophageal cancer corridor. On the contrary, western Sub-Saharan Africa was shown to be the cold spot of esophageal cancer in comparison with other counterparts. Only approximately 0.25% of the Sub-Saharan Africa population is covered by accurate death registration systems (12). Due to the rarity and inaccessibility of the diagnostic means, the epidemic data in sub-Saharan Africa are fragmented and not numerous. Risk factors for esophageal cancer have not been well-documented in Sub-Saharan Africa.

Previous researches have demonstrated a negative correlation between socioeconomic levels and ESCC (13, 14). A nationwide Swedish case-control study also demonstrated a relationship of EAC and low socioeconomic status (15). Indeed, we saw an inverse association between SDI and ASRs of esophageal cancer. The ASRs in East Asia and Southern Sub-Saharan Africa were higher than expected from 1990 to 2019. Therefore, regional trends in ASRs should not just be considered in isolation, and their observed values should be compared to expected values to evaluate the management of esophageal cancer.

It is important to apply a life table approach to assess differences in exposure and hereditary susceptibility between females and males. The esophageal cancer burden in male was heavier than females. Globally, the ASRs in females decreased more than those in males from 1990 to 2019. This pattern is similar with other worldwide studies (1, 16). The reasons for the large gender gap in esophageal cancer are not completely understood. The differences in prevalence of gastroesophageal reflux disease, obesity, smoking and other risk factors between males and females should be considered. The difference of sex steroid may be another cause of that sex difference. Evidence from murine model suggests a promoting effect of androgen and an inhibiting effect of estrogen on the experimental induction of esophageal cancer (17). A pooled analysis of case-control studies also found that breastfeeding could decrease the risk of esophageal cancer (18). In addition, females are more likely seek healthcare at earlier cancer stage, and have a better prognosis than males (19). Esophageal cancer is typically a disease of the elderly, as more than 80% of newly diagnosed patients are over the age of 55 (2), with most in their 60s and 70s. With the rapid aging of the population, the number of older patients is increasing. The death is also closely related to age. Elderly patients often not adequately treated even for curable stages of the esophageal cancer (20). Compared to the younger patients with esophageal cancer, elderly patients have more perioperative complications and shorter overall survival from oesophagectomy (21–23).

Based on previous studies, the risk of esophageal cancer is considerably higher in current and former smokers, compared with those who had never smoked (24–27). The major risk factor of ESCC in high-income countries is cigarette smoking. The deaths of esophageal cancer attributable to smoking decreased between 1990 and 2019 in high SDI countries, but it was still not lower than the proportion of esophageal cancer related deaths attributable to alcohol use and high BMI in 2019. This finding is consistent with the reductions in smoking in high-income countries. Since 1990, the age-standardized prevalence of current smoking has decreased by 32.2% for males and 28.8% for females in high-income countries (28).

Alcohol is clearly linked to increase the risk of ESCC (2, 4, 24, 26, 29). In contrast, most studies have not shown a significant collection between alcohol and EAC (2, 4, 24–26, 30). However, some studies tend to support an increased risk of EAC in heavy alcohol drinking (31, 32). The increase in risk is dependent on the type and amount of alcohol consumed (2). Present study estimated that 22.6% of the esophageal cancer deaths were attributable to alcohol, and the percentage of alcohol use increased in most countries from 1990 to 2019. The increases in the proportion of esophageal cancer deaths attributable to alcohol are similar to worldwide increases in alcohol consumption. The adult per-capita consumption increased from 5.9 L in 1990 to 6.5 L in 2017, and will reach 7.6 L by 2030 (33). Simultaneously, the prevalence of current drinking increased from 45 to 47% between 1990 and 2017, and is expected to reach 50% by 2030 (33). Therefore, the impact of alcohol on esophageal cancer is expected to be even greater in the future.

High BMI is another risk factor for EAC (2, 24, 29, 34, 35). As shown in a meta-analysis of 141 studies, each 5 kg/m^2^ increase in BMI is strongly associated with a significant increase in risk for EAC (35). It has been postulated that obesity would presumably increase Barrett's esophagus by increasing the risk of hiatal hernia and gastroesophageal reflux (36, 37), which in turn is a primary risk factor for EAC. In addition, adipose tissue itself affects tumor development by a specific mechanism (38). Interestingly, a higher BMI relates with a lower risk for ESCC (2, 34, 39–41). The mechanism by which high BMI might decrease ESCC remains unknown. It seems that those patients with low BMI tend to be malnourished, suggesting they may have a micronutrient deficiency that raises the risk of ESCC. At the same time, drinker and smoker often have a low BMI, and as stated above, alcohol and tobacco use increase the risks of ESCC. We found that the contribution of high BMI increased globally, which is consistent with the increasing prevalence of obesity across the world, as well as the increases in the incidence of EAC (1, 2).

Most of studies have found that the significance of fruits and vegetables intake is not only a non-specific sign of a well-balanced diet but it also has a protective effect against esophageal cancer (2, 4, 29, 42, 43). Fruits and vegetables add macro and micro nutrients, as well as dietary fiber and phytochemicals to provide health gaining benefits (44). Variety in vegetable or fruit consumption decreases the risk of ESCC while no effect is seen for EAC (45). The debate about the mechanism by which fruits and vegetables might exert their protective effect is still on. The burden of esophageal cancer related to diet low in vegetables and fruits were highest in low and low-middle SDI countries. The prevalence of diet low in vegetables and fruits tends to decrease with income (29). Individual with higher income and education are unlikely to have problems with low fruit and vegetable consumption. The deaths of esophageal cancer attributable to diet low in vegetables and diet low in fruits decreased globally between 1990 and 2019, but it tended to stay stable in low SDI countries. In this light, policymakers, especially in low and low-middle SDI countries, are required to promote the maintenance of well-balanced diet.

There exist some unavoidable limitations in this study. First, several countries, especially low-income countries, did not have any high-quality data, and hence their estimates were based on the modeling in this occasion. Second, the epidemiological features, outcomes and risk factors are quite different between ESCC and EAC, but data for two subtypes did not been distinguished in our study. Third, other risk factors did not include in GBD study, such as hot drinks, genetics and family history. Lastly, the fluctuations in incidence and death may partly reflect testing bias related to changes in screening programs rather than actual changes in age-specific rates. Further studies should be conducted to provide a comprehensive evaluation.

## Conclusion

Our study found that the burden of esophageal cancer was heterogeneous across regions and countries by sex, age, and SDI. Although the global ASIR, ASDR, and age-standardized DALY rate had decreased between 1990 and 2019, esophageal cancer still was an important burden in some high-risk areas. Steps against attributable risk factors may be effective in reducing the burden of esophageal cancer. Furthermore, geographic differences in the burden should not be ignored in allocating limited resources and formulating relevant policies. Further researches are required to expand our knowledge of additional factors associated with esophageal cancer incidence and to formulate more detailed prevention and intervention strategies, especially in high-risk areas. Meanwhile, efforts to improve data collection and sharing, especially in low SDI countries, should be taken into account in future researches.

## Data availability statement

The datasets presented in this study can be found in online repositories. The names of the repository/repositories and accession number(s) can be found below: http://ghdx.healthdata.org/gbd-results-tool.

## Author contributions

YC: study design, data collection and analysis, interpretation of results, and writing–original draft. JL: interpretation of results and writing–original draft. WW: interpretation of results and writing–review and editing. PC: data collection, analysis, and writing–review and editing. KY: study design, interpretation of results, and writing–review and editing. All authors contributed to the article and approved the submitted version.

## Conflict of interest

The authors declare that the research was conducted in the absence of any commercial or financial relationships that could be construed as a potential conflict of interest.

## Publisher's note

All claims expressed in this article are solely those of the authors and do not necessarily represent those of their affiliated organizations, or those of the publisher, the editors and the reviewers. Any product that may be evaluated in this article, or claim that may be made by its manufacturer, is not guaranteed or endorsed by the publisher.
