# Supplementary material for: Burden of esophageal cancer and its attributable risk factors in 204 countries and territories from 1990 to 2019
Source: Front Public Health. 2022 Sep 6;10:952087. doi: 10.3389/fpubh.2022.952087 (PMC9485842; doi:10.3389/fpubh.2022.952087)
Supplement: Supplementary file 1 [file Table_1.docx]

**Supplementary Table 1. Esophageal incidence cases, age-standardized incidence rate, deaths, age-standardized death rate,** **disability-adjusted life-years, and age-standardized disability-adjusted life-years rate in 2019.**

| **Characteristics** | **Incidence cases (95%UI)** | **ASIR per 10^5^ (95% UI)** | **Deaths (95% UI)** | **ASMR per 10^5^ (95% UI)** | **DALYs (95% UI)** | **Age-standardized DALY rates per 10^5^ (95% UI)** |
| --- | --- | --- | --- | --- | --- | --- |
| Afghanistan | 827(266-1203) | 6.55(2.11-9.17) | 832(268-1202) | 6.96(2.25-9.70) | 24997(8044-37747) | 169.51(54.59-243.26) |
| Albania | 59(42-86) | 1.38(1.00-2.01) | 61(44-88) | 1.44(1.05-2.06) | 1369(965-2053) | 33.48(23.77-49.54) |
| Algeria | 344(244-439) | 1.08(0.79-1.37) | 349(251-446) | 1.15(0.85-1.44) | 8402(5895-10838) | 24.24(17.20-31.06) |
| American Samoa | 1(1-1) | 1.42(1.11-1.68) | 1(1-1) | 1.52(1.18-1.78) | 17(13-20) | 33.73(26.26-40.50) |
| Andorra | 6(4-8) | 4.15(3.02-5.36) | 5(4-6) | 3.56(2.63-4.55) | 121(88-157) | 86.70(62.82-112.65) |
| Angola | 908(498-1221) | 8.24(4.44-11.10) | 920(502-1238) | 8.81(4.73-11.89) | 26327(14487-35620) | 209.13(113.76-281.53) |
| Antigua and Barbuda | 3(3-4) | 2.98(2.53-3.54) | 3(3-4) | 3.11(2.64-3.66) | 71(59-85) | 67.89(56.79-80.64) |
| Argentina | 2681(2121-3366) | 4.97(3.93-6.25) | 2759(2527-3007) | 5.08(4.66-5.53) | 59142(54358-64209) | 112.09(103.21-121.83) |
| Armenia | 64(52-77) | 1.53(1.26-1.84) | 67(55-81) | 1.62(1.34-1.94) | 1523(1242-1845) | 36.43(29.74-44.00) |
| Australia | 1815(1396-2342) | 4.34(3.34-5.61) | 1720(1525-1905) | 4.03(3.61-4.45) | 33736(30354-37061) | 85.76(77.85-94.00) |
| Austria | 501(400-622) | 3.02(2.40-3.77) | 425(385-468) | 2.49(2.26-2.73) | 9477(8653-10369) | 60.82(55.46-66.81) |
| Azerbaijan | 781(595-1188) | 8.67(6.49-13.36) | 796(601-1220) | 9.32(6.87-14.35) | 21021(16036-31587) | 208.98(159.15-316.11) |
| Bahamas | 20(16-25) | 5.06(4.03-6.28) | 20(16-25) | 5.19(4.15-6.40) | 550(434-691) | 129.88(103.11-163.11) |
| Bahrain | 16(12-22) | 1.94(1.45-2.48) | 16(11-21) | 2.03(1.52-2.57) | 428(307-577) | 39.70(29.24-51.88) |
| Bangladesh | 4982(3522-7507) | 3.81(2.71-5.73) | 5110(3629-7680) | 4.00(2.87-6.00) | 132618(92755-200855) | 97.07(68.50-145.98) |
| Barbados | 24(19-29) | 4.92(3.97-5.96) | 25(20-30) | 5.09(4.14-6.13) | 553(443-681) | 115.47(92.42-142.45) |
| Belarus | 483(366-639) | 3.05(2.31-4.04) | 471(357-620) | 2.96(2.24-3.91) | 12686(9522-16926) | 81.97(62.05-109.54) |
| Belgium | 1129(883-1432) | 5.26(4.04-6.68) | 1007(913-1101) | 4.52(4.12-4.93) | 21196(19401-23073) | 106.29(97.53-115.67) |
| Belize | 7(6-8) | 2.38(2.03-2.77) | 7(6-8) | 2.46(2.10-2.85) | 184(156-215) | 60.81(51.68-70.78) |
| Benin | 233(139-321) | 4.86(2.88-6.63) | 240(142-330) | 5.18(3.05-6.99) | 6439(3884-9041) | 122.93(73.05-171.41) |
| Bermuda | 6(5-7) | 4.44(3.67-5.46) | 6(5-7) | 4.26(3.52-5.21) | 118(96-146) | 96.24(78.47-118.60) |
| Bhutan | 26(18-36) | 4.69(3.30-6.46) | 27(19-37) | 5.00(3.56-6.86) | 672(456-949) | 114.53(78.75-161.67) |
| Bolivia  (Plurinational State of) | 195(150-247) | 2.33(1.82-2.92) | 211(163-265) | 2.59(2.03-3.22) | 4705(3595-6077) | 52.82(40.55-67.46) |
| Bosnia and Herzegovina | 120(92-151) | 2.01(1.56-2.54) | 123(95-155) | 2.06(1.61-2.60) | 2943(2258-3748) | 50.37(38.69-64.08) |
| Botswana | 178(110-241) | 12.72(7.73-16.72) | 177(109-238) | 13.21(8.08-17.32) | 5110(3081-7088) | 328.37(201.96-443.72) |
| Brazil | 12473(11792-13087) | 5.20(4.90-5.45) | 12551(11802-13222) | 5.28(4.95-5.56) | 323233(306838-340084) | 132.00(125.20-138.86) |
| Brunei Darussalam | 9(7-11) | 3.25(2.76-3.99) | 8(6-9) | 3.03(2.57-3.73) | 196(165-241) | 62.31(52.83-76.60) |
| Bulgaria | 258(201-327) | 1.98(1.53-2.50) | 261(203-329) | 1.95(1.51-2.46) | 6537(5044-8300) | 53.52(41.29-68.51) |
| Burkina Faso | 431(252-558) | 4.78(2.78-6.12) | 443(259-573) | 5.09(2.96-6.48) | 12012(7063-15592) | 121.44(70.96-156.76) |
| Burundi | 538(358-783) | 11.49(7.73-16.36) | 546(363-786) | 12.18(8.18-17.18) | 15905(10483-23309) | 304.13(202.23-438.41) |
| Cabo Verde | 65(54-77) | 15.57(13.02-18.14) | 68(57-79) | 16.38(13.70-18.91) | 1699(1396-2052) | 388.77(320.50-464.13) |
| Cambodia | 333(255-460) | 2.82(2.17-3.89) | 339(261-467) | 2.97(2.30-4.10) | 8988(6717-12384) | 70.44(53.61-97.28) |
| Cameroon | 668(371-962) | 5.64(3.10-7.97) | 682(377-977) | 5.98(3.28-8.40) | 18844(10435-27426) | 144.06(79.90-207.75) |
| Canada | 3002(2347-3792) | 4.42(3.44-5.59) | 2527(2265-2763) | 3.64(3.27-3.98) | 52497(47296-57562) | 80.92(73.01-88.47) |
| Central African Republic | 231(130-323) | 10.28(5.74-14.14) | 234(132-327) | 10.95(6.14-14.94) | 7129(4032-10191) | 280.00(158.10-390.36) |
| Chad | 256(146-352) | 4.64(2.64-6.33) | 265(150-362) | 4.95(2.82-6.72) | 7106(4073-9751) | 118.23(67.15-162.40) |
| Chile | 935(728-1178) | 3.87(3.02-4.89) | 964(854-1065) | 4.00(3.54-4.42) | 17184(15485-18789) | 71.23(64.26-77.93) |
| China | 278121(213512-331600) | 13.90(10.70-16.52) | 257316(202777-309029) | 13.15(10.27-15.68) | 5759997(4581771-6999574) | 277.50(221.69-335.87) |
| Colombia | 1013(767-1308) | 1.91(1.44-2.47) | 1065(812-1372) | 2.00(1.52-2.58) | 21716(16272-28144) | 41.21(30.81-53.53) |
| Comoros | 55(37-77) | 11.25(7.65-15.77) | 57(39-79) | 11.94(8.15-16.62) | 1485(974-2121) | 289.87(193.97-408.21) |
| Congo | 260(145-359) | 10.05(5.57-13.71) | 264(148-362) | 10.76(5.96-14.53) | 7463(4185-10401) | 254.97(142.76-348.11) |
| Cook Islands | 1(1-1) | 2.65(2.20-3.18) | 1(1-1) | 2.67(2.24-3.18) | 15(12-18) | 60.20(49.08-73.72) |
| Costa Rica | 88(67-112) | 1.73(1.32-2.22) | 91(69-116) | 1.79(1.37-2.28) | 1902(1452-2459) | 36.89(28.26-47.78) |
| Croatia | 531(298-738) | 5.04(2.79-6.84) | 538(300-743) | 5.35(2.95-7.25) | 15190(8473-21441) | 127.77(71.40-176.79) |
| Cuba | 230(174-296) | 2.77(2.10-3.61) | 220(168-283) | 2.62(1.99-3.38) | 5126(3839-6702) | 66.23(49.81-86.43) |
| Cyprus | 985(783-1219) | 5.21(4.14-6.46) | 966(770-1191) | 5.08(4.05-6.28) | 23797(18771-29687) | 128.22(100.97-159.65) |
| Czechia | 27(21-33) | 1.41(1.08-1.69) | 24(18-29) | 1.27(0.97-1.50) | 509(389-608) | 26.58(20.53-31.78) |
| Côte d'Ivoire | 660(528-816) | 3.36(2.68-4.19) | 619(496-764) | 3.09(2.48-3.85) | 14596(11652-18256) | 78.72(62.69-99.23) |
| Democratic People's Republic of Korea | 2923(2253-3771) | 8.96(6.96-11.60) | 2906(2259-3752) | 9.01(7.06-11.56) | 75140(56792-99032) | 225.46(171.56-293.98) |
| Democratic Republic of the Congo | 2884(1530-4156) | 8.17(4.29-11.95) | 2938(1560-4244) | 8.71(4.60-12.77) | 82506(43849-118954) | 208.75(111.04-301.08) |
| Denmark | 562(435-720) | 5.01(3.88-6.50) | 517(460-581) | 4.50(4.00-5.04) | 10572(9424-11883) | 100.19(89.57-112.68) |
| Djibouti | 69(43-111) | 11.37(7.36-17.27) | 70(44-112) | 12.13(7.90-18.30) | 2063(1243-3387) | 295.01(185.34-466.56) |
| Dominica | 4(4-5) | 4.87(3.91-6.02) | 5(4-6) | 5.14(4.14-6.32) | 106(84-133) | 118.91(94.26-149.49) |
| Dominican Republic | 226(155-316) | 2.43(1.68-3.38) | 234(162-324) | 2.55(1.79-3.52) | 5959(3940-8385) | 61.79(41.25-86.92) |
| Ecuador | 220(169-298) | 1.52(1.18-2.03) | 238(184-317) | 1.68(1.30-2.21) | 4936(3723-6918) | 32.61(24.80-45.15) |
| Egypt | 987(670-1318) | 1.50(1.04-2.01) | 968(661-1286) | 1.54(1.06-2.09) | 27880(18878-37726) | 38.56(26.26-51.55) |
| El Salvador | 95(72-122) | 1.59(1.20-2.06) | 102(78-131) | 1.68(1.28-2.17) | 2144(1610-2795) | 36.48(27.29-47.79) |
| Equatorial Guinea | 39(20-69) | 8.49(4.42-14.49) | 40(21-71) | 9.20(4.77-15.56) | 1058(550-1941) | 205.75(107.59-368.78) |
| Eritrea | 361(226-505) | 13.20(8.37-18.14) | 361(227-505) | 13.92(8.85-18.99) | 10887(6787-15400) | 347.49(218.60-484.46) |
| Estonia | 71(53-91) | 2.98(2.24-3.83) | 71(54-91) | 2.91(2.19-3.74) | 1677(1254-2157) | 75.44(56.04-97.76) |
| Eswatini | 89(57-123) | 15.14(9.70-20.59) | 91(58-124) | 15.86(10.18-21.40) | 2623(1630-3707) | 409.37(259.15-568.22) |
| Ethiopia | 1079(852-1505) | 2.67(2.11-3.72) | 1119(869-1569) | 2.87(2.24-4.03) | 29766(22793-41213) | 67.11(51.79-93.43) |
| Fiji | 19(10-25) | 2.67(1.51-3.45) | 19(11-25) | 2.90(1.65-3.72) | 501(279-666) | 63.74(35.67-83.51) |
| Finland | 382(289-488) | 3.21(2.44-4.14) | 309(274-345) | 2.50(2.23-2.79) | 6011(5369-6742) | 55.00(48.94-61.72) |
| France | 5813(4539-7406) | 4.62(3.58-5.95) | 5216(4672-5765) | 3.96(3.55-4.35) | 108985(97755-120332) | 94.39(84.97-104.12) |
| Gabon | 110(63-148) | 10.56(6.01-13.93) | 113(64-150) | 11.22(6.38-14.63) | 3027(1730-4132) | 266.17(151.64-358.94) |
| Gambia | 20(15-25) | 2.09(1.57-2.66) | 20(15-26) | 2.22(1.67-2.79) | 540(388-707) | 53.43(39.08-69.59) |
| Georgia | 104(85-126) | 1.80(1.47-2.17) | 109(89-130) | 1.85(1.52-2.22) | 2633(2127-3176) | 47.57(38.48-57.38) |
| Germany | 9602(7438-12502) | 5.48(4.24-7.20) | 6746(6150-7413) | 3.71(3.39-4.07) | 145808(133236-159444) | 90.01(82.30-98.45) |
| Ghana | 524(365-686) | 3.32(2.29-4.30) | 538(373-703) | 3.52(2.42-4.54) | 14332(10117-19029) | 83.01(57.60-108.61) |
| Greece | 355(277-451) | 1.57(1.22-2.00) | 333(301-367) | 1.39(1.26-1.52) | 6320(5765-6908) | 31.68(28.97-34.47) |
| Greenland | 9(7-11) | 12.96(10.41-15.38) | 9(7-11) | 13.13(10.43-15.69) | 235(183-286) | 309.62(242.93-374.86) |
| Grenada | 7(7-8) | 6.48(5.68-7.32) | 8(7-9) | 6.66(5.85-7.56) | 193(168-220) | 162.42(141.81-184.13) |
| Guam | 5(4-6) | 2.61(2.05-3.20) | 5(4-6) | 2.65(2.08-3.22) | 125(98-155) | 64.96(50.46-80.48) |
| Guatemala | 222(173-281) | 2.05(1.61-2.59) | 235(184-296) | 2.23(1.77-2.80) | 5533(4260-7084) | 47.89(37.08-61.29) |
| Guinea | 104(78-135) | 1.91(1.44-2.45) | 108(81-139) | 2.02(1.54-2.57) | 2867(2123-3744) | 49.14(36.64-63.65) |
| Guinea-Bissau | 45(25-61) | 6.19(3.41-8.26) | 45(25-61) | 6.42(3.55-8.59) | 1293(713-1790) | 160.51(88.36-217.23) |
| Guyana | 14(10-18) | 2.09(1.61-2.69) | 14(10-18) | 2.18(1.68-2.77) | 380(284-498) | 55.08(41.91-71.57) |
| Haiti | 244(161-364) | 3.52(2.37-5.19) | 249(165-369) | 3.75(2.55-5.46) | 6835(4463-10316) | 89.86(59.46-134.76) |
| Honduras | 97(68-127) | 1.66(1.17-2.16) | 102(72-133) | 1.80(1.27-2.32) | 2416(1664-3208) | 39.32(27.29-52.00) |
| Hungary | 652(523-800) | 3.73(2.97-4.62) | 643(521-785) | 3.61(2.89-4.46) | 16428(13086-20396) | 98.92(77.87-124.12) |
| Iceland | 26(22-31) | 4.87(4.14-5.73) | 22(19-25) | 3.92(3.41-4.51) | 469(409-540) | 91.51(79.98-105.30) |
| India | 38516(31813-55988) | 3.36(2.78-4.89) | 38981(32203-56283) | 3.49(2.90-5.05) | 1055009(869421-1514411) | 86.99(71.98-124.54) |
| Indonesia | 4989(3956-7218) | 2.36(1.89-3.41) | 5069(4062-7233) | 2.51(2.02-3.58) | 133605(106643-191026) | 57.40(46.08-81.45) |
| Iran  (Islamic Republic of) | 3031(2417-3332) | 4.36(3.43-4.80) | 3069(2420-3361) | 4.54(3.54-4.99) | 70307(57445-77012) | 94.25(75.29-103.27) |
| Iraq | 291(221-368) | 1.26(0.97-1.55) | 286(219-358) | 1.30(1.01-1.59) | 7966(5928-10256) | 30.97(23.57-39.16) |
| Ireland | 570(436-736) | 7.61(5.82-9.89) | 485(421-554) | 6.41(5.59-7.31) | 9704(8461-11120) | 133.54(116.90-152.53) |
| Israel | 189(145-240) | 1.62(1.24-2.08) | 182(159-204) | 1.53(1.35-1.71) | 3404(3031-3784) | 30.58(27.42-33.92) |
| Italy | 2812(2286-3365) | 2.05(1.67-2.48) | 2635(2394-2829) | 1.83(1.69-1.96) | 50605(47287-54137) | 41.12(38.69-44.02) |
| Jamaica | 77(59-97) | 2.59(2.00-3.30) | 80(62-102) | 2.70(2.10-3.43) | 1793(1361-2308) | 60.73(46.19-78.20) |
| Japan | 21521(17654-25788) | 6.45(5.29-7.79) | 14000(12469-15103) | 3.91(3.59-4.19) | 255594(235520-274033) | 85.81(80.47-91.96) |
| Jordan | 73(58-91) | 1.14(0.93-1.41) | 71(56-87) | 1.16(0.95-1.43) | 1892(1462-2355) | 26.16(20.79-32.39) |
| Kazakhstan | 1238(1052-1447) | 7.35(6.27-8.51) | 1276(1088-1482) | 7.80(6.67-9.02) | 31017(26091-36787) | 173.54(146.82-203.35) |
| Kenya | 2576(1935-3623) | 11.93(8.96-16.74) | 2831(2156-4035) | 13.72(10.54-19.58) | 77800(58838-111602) | 320.86(244.53-458.05) |
| Kiribati | 5(2-7) | 7.66(3.30-9.94) | 5(2-7) | 8.23(3.55-10.56) | 159(64-216) | 201.47(83.53-265.92) |
| Kuwait | 35(28-43) | 1.50(1.19-1.87) | 33(27-41) | 1.53(1.22-1.90) | 803(641-1005) | 28.94(23.11-35.77) |
| Kyrgyzstan | 179(152-208) | 4.13(3.51-4.82) | 186(158-217) | 4.45(3.79-5.17) | 4608(3881-5396) | 98.42(83.48-115.05) |
| Lao People's Democratic Republic | 104(71-151) | 2.39(1.66-3.50) | 106(73-155) | 2.53(1.77-3.69) | 2917(1956-4193) | 60.51(41.43-88.11) |
| Latvia | 118(91-151) | 3.30(2.54-4.28) | 120(94-154) | 3.30(2.55-4.25) | 2954(2264-3841) | 88.54(67.19-116.58) |
| Lebanon | 68(51-89) | 1.30(0.97-1.72) | 65(49-86) | 1.25(0.94-1.66) | 1447(1075-1899) | 27.83(20.63-36.68) |
| Lesotho | 188(120-253) | 14.59(9.17-19.34) | 193(123-258) | 15.44(9.65-20.36) | 5506(3453-7539) | 398.10(253.46-540.74) |
| Liberia | 98(55-136) | 4.88(2.69-6.74) | 101(56-139) | 5.21(2.87-7.15) | 2766(1545-3911) | 122.55(68.27-170.99) |
| Libya | 70(49-90) | 1.37(0.97-1.75) | 70(49-90) | 1.41(1.01-1.80) | 1862(1276-2449) | 33.38(23.27-43.41) |
| Lithuania | 195(155-243) | 3.79(2.99-4.75) | 193(153-240) | 3.68(2.92-4.61) | 4870(3846-6144) | 100.56(78.55-127.42) |
| Luxembourg | 38(30-47) | 3.84(3.07-4.86) | 33(28-40) | 3.34(2.83-4.00) | 742(627-897) | 78.31(66.06-94.92) |
| Madagascar | 1100(689-1557) | 9.74(6.18-13.64) | 1108(698-1557) | 10.31(6.61-14.36) | 33071(20747-46479) | 257.48(162.44-361.88) |
| Malawi | 1810(1346-2438) | 24.53(18.74-32.51) | 1843(1382-2468) | 25.76(19.76-33.94) | 52484(38093-72014) | 651.57(481.63-882.94) |
| Malaysia | 690(531-871) | 2.66(2.06-3.34) | 678(525-856) | 2.70(2.10-3.36) | 16320(12560-20857) | 59.09(45.73-75.08) |
| Maldives | 6(5-7) | 2.05(1.65-2.48) | 6(5-7) | 2.01(1.62-2.44) | 138(111-168) | 42.24(33.89-51.63) |
| Mali | 220(165-295) | 2.55(1.95-3.37) | 225(170-299) | 2.69(2.08-3.53) | 6183(4561-8421) | 66.07(49.35-88.60) |
| Malta | 22(18-27) | 2.50(2.04-3.06) | 19(16-23) | 2.15(1.81-2.54) | 409(345-482) | 50.26(42.84-59.17) |
| Marshall Islands | 1(1-1) | 2.68(1.86-3.73) | 1(1-1) | 2.87(2.00-3.93) | 27(18-38) | 67.77(46.05-95.94) |
| Mauritania | 84(49-113) | 4.14(2.44-5.53) | 88(52-117) | 4.46(2.64-5.91) | 2148(1277-2990) | 99.98(59.51-136.96) |
| Mauritius | 44(35-56) | 2.53(2.03-3.18) | 43(35-54) | 2.52(2.02-3.15) | 1043(830-1330) | 58.39(46.42-74.36) |
| Mexico | 1668(1417-1962) | 1.44(1.23-1.69) | 1720(1456-2008) | 1.51(1.28-1.75) | 40458(33947-47718) | 33.75(28.42-39.73) |
| Micronesia  (Federated States of) | 2(1-3) | 3.04(2.10-4.17) | 2(1-3) | 3.23(2.26-4.39) | 61(39-87) | 77.08(50.97-107.55) |
| Monaco | 6(5-7) | 6.55(4.95-8.06) | 5(4-6) | 5.27(4.07-6.45) | 97(74-119) | 119.31(90.27-147.59) |
| Mongolia | 426(327-550) | 21.93(14.53-27.95) | 448(333-577) | 24.53(15.33-31.27) | 10863(8385-14155) | 486.69(364.21-628.73) |
| Montenegro | 23(18-29) | 2.40(1.90-2.98) | 23(18-29) | 2.37(1.88-2.93) | 595(467-745) | 62.13(48.72-77.71) |
| Morocco | 485(355-599) | 1.57(1.18-1.90) | 492(365-600) | 1.65(1.26-1.99) | 12461(9015-15741) | 37.75(27.57-46.86) |
| Mozambique | 834(617-1115) | 7.80(5.76-10.31) | 864(644-1151) | 8.37(6.12-11.08) | 23518(17342-31906) | 200.37(148.04-267.75) |
| Myanmar | 1039(816-1577) | 2.25(1.79-3.39) | 1060(839-1604) | 2.37(1.90-3.58) | 27994(21666-41721) | 56.63(44.30-85.47) |
| Namibia | 38(28-49) | 2.63(2.03-3.36) | 38(29-49) | 2.74(2.14-3.46) | 1054(771-1412) | 69.56(51.85-91.49) |
| Nauru | 0(0-0) | 3.14(2.19-4.16) | 0(0-0) | 3.31(2.35-4.32) | 4(3-6) | 78.45(53.16-106.07) |
| Nepal | 1053(772-1428) | 4.78(3.55-6.48) | 1091(804-1480) | 5.10(3.81-6.80) | 28099(20397-38410) | 120.24(87.93-163.27) |
| Netherlands | 2810(2175-3531) | 8.37(6.47-10.58) | 2399(2164-2638) | 6.98(6.34-7.64) | 48594(44278-53139) | 152.58(139.01-166.59) |
| New Zealand | 376(303-456) | 4.79(3.86-5.79) | 315(281-348) | 3.93(3.53-4.33) | 6150(5575-6716) | 82.16(75.04-89.60) |
| Nicaragua | 50(39-64) | 1.20(0.94-1.55) | 53(42-68) | 1.32(1.06-1.70) | 1182(926-1529) | 26.62(20.83-34.40) |
| Niger | 292(169-405) | 3.86(2.24-5.22) | 300(174-415) | 4.15(2.40-5.56) | 8055(4699-11377) | 96.40(56.00-133.23) |
| Nigeria | 743(514-1342) | 0.91(0.65-1.58) | 780(536-1419) | 1.00(0.71-1.76) | 20018(13380-38073) | 21.87(14.89-40.04) |
| Niue | 0(0-0) | 2.46(1.88-3.01) | 0(0-0) | 2.52(1.92-3.05) | 1(1-2) | 57.37(42.87-71.80) |
| North Macedonia | 49(38-62) | 1.52(1.19-1.93) | 49(38-63) | 1.56(1.23-1.97) | 1246(956-1613) | 38.64(29.71-49.93) |
| Northern Mariana Islands | 1(1-2) | 2.95(2.10-3.52) | 1(1-2) | 3.01(2.12-3.55) | 38(27-47) | 66.51(47.20-80.20) |
| Norway | 268(224-320) | 2.85(2.37-3.40) | 236(217-257) | 2.44(2.26-2.65) | 4736(4407-5154) | 53.07(49.50-57.82) |
| Oman | 37(27-47) | 2.37(1.84-2.79) | 34(25-43) | 2.38(1.87-2.78) | 958(684-1258) | 49.78(37.50-60.66) |
| Pakistan | 8911(7068-10991) | 7.86(6.30-9.63) | 8953(7184-11011) | 8.23(6.62-9.96) | 260192(204466-323900) | 207.08(165.90-255.39) |
| Palau | 1(0-1) | 2.45(1.95-3.08) | 0(0-1) | 2.51(2.00-3.12) | 14(10-18) | 58.72(45.73-74.81) |
| Palestine | 25(20-33) | 1.10(0.90-1.49) | 25(20-33) | 1.16(0.94-1.58) | 649(527-837) | 25.53(20.70-33.63) |
| Panama | 66(49-86) | 1.60(1.19-2.07) | 68(51-88) | 1.64(1.24-2.12) | 1534(1134-2009) | 36.99(27.36-48.31) |
| Papua New Guinea | 86(61-126) | 1.88(1.36-2.81) | 86(62-126) | 2.02(1.47-3.04) | 2494(1758-3671) | 46.82(33.55-68.80) |
| Paraguay | 210(155-276) | 3.83(2.84-5.03) | 217(160-284) | 3.99(2.96-5.22) | 5197(3775-6853) | 91.50(66.79-120.23) |
| Peru | 412(306-542) | 1.29(0.96-1.70) | 440(329-574) | 1.38(1.03-1.80) | 9199(6794-12222) | 28.57(21.10-37.94) |
| Philippines | 1052(837-1402) | 1.32(1.06-1.75) | 1046(840-1418) | 1.36(1.10-1.88) | 29414(23476-39162) | 34.07(27.34-45.60) |
| Poland | 2144(1754-2610) | 3.19(2.60-3.91) | 2233(1834-2706) | 3.28(2.70-3.99) | 53106(43145-65194) | 83.00(67.20-102.14) |
| Portugal | 730(561-950) | 3.47(2.61-4.55) | 691(621-762) | 3.17(2.86-3.50) | 15983(14457-17656) | 83.51(75.43-92.22) |
| Puerto Rico | 178(137-229) | 2.56(1.96-3.31) | 180(140-231) | 2.50(1.92-3.22) | 3651(2770-4740) | 56.90(43.06-74.22) |
| Qatar | 24(16-35) | 4.47(3.16-6.14) | 21(14-31) | 4.84(3.38-6.65) | 631(416-990) | 75.55(53.50-103.88) |
| Republic of Korea | 3419(2691-4689) | 3.76(2.96-5.16) | 2193(1891-3173) | 2.43(2.09-3.52) | 47390(40428-70394) | 51.74(44.19-76.96) |
| Republic of Moldova | 104(87-124) | 1.79(1.51-2.13) | 103(87-123) | 1.78(1.50-2.11) | 2844(2380-3386) | 49.88(41.80-59.33) |
| Romania | 862(695-1041) | 2.59(2.07-3.15) | 855(692-1033) | 2.52(2.02-3.06) | 22725(18077-27625) | 71.70(56.38-87.81) |
| Russian Federation | 7953(6736-9370) | 3.38(2.86-3.98) | 7663(6457-8935) | 3.24(2.73-3.79) | 194853(163024-229953) | 84.60(70.65-100.06) |
| Rwanda | 650(429-932) | 10.80(7.10-15.23) | 663(438-948) | 11.50(7.66-16.11) | 18432(11944-26809) | 276.42(182.61-395.85) |
| Saint Kitts and Nevis | 3(2-4) | 4.33(3.60-5.14) | 3(2-3) | 4.36(3.67-5.15) | 76(59-92) | 103.57(82.80-124.74) |
| Saint Lucia | 9(8-11) | 4.32(3.58-5.15) | 10(8-11) | 4.47(3.70-5.31) | 240(198-287) | 108.17(89.08-129.60) |
| Saint Vincent and the Grenadines | 3(3-4) | 2.38(2.04-2.77) | 3(3-4) | 2.48(2.13-2.87) | 84(71-99) | 60.66(51.55-71.06) |
| Samoa | 2(2-3) | 1.61(1.22-2.01) | 2(2-3) | 1.68(1.29-2.08) | 60(43-78) | 39.37(28.81-50.14) |
| San Marino | 1(1-1) | 1.71(1.28-2.24) | 1(1-1) | 1.47(0.97-2.09) | 19(12-28) | 34.63(21.61-51.15) |
| Sao Tome and Principe | 5(3-6) | 4.81(2.63-6.03) | 5(3-6) | 5.13(2.77-6.43) | 135(74-176) | 119.79(65.20-153.05) |
| Saudi Arabia | 313(235-433) | 1.78(1.38-2.36) | 287(215-394) | 1.78(1.40-2.36) | 8787(6446-12667) | 39.79(30.40-53.62) |
| Senegal | 344(192-460) | 4.68(2.60-6.21) | 358(199-476) | 5.01(2.78-6.60) | 9221(5128-12497) | 116.82(65.21-156.85) |
| Serbia | 367(285-469) | 2.43(1.88-3.12) | 375(292-475) | 2.45(1.91-3.14) | 9142(6999-11852) | 63.41(48.34-81.91) |
| Seychelles | 6(5-8) | 5.63(4.76-6.67) | 6(5-7) | 5.61(4.76-6.66) | 168(141-201) | 140.57(117.98-167.78) |
| Sierra Leone | 152(84-208) | 4.32(2.35-5.83) | 158(86-215) | 4.60(2.51-6.21) | 4192(2297-5800) | 109.48(59.88-150.74) |
| Singapore | 210(162-269) | 2.71(2.12-3.45) | 137(120-160) | 1.81(1.57-2.10) | 2938(2574-3432) | 36.74(32.20-42.92) |
| Slovakia | 313(223-416) | 3.48(2.49-4.62) | 289(206-382) | 3.19(2.29-4.22) | 7495(5323-10012) | 85.57(60.76-113.99) |
| Slovenia | 116(88-152) | 2.85(2.15-3.76) | 105(79-137) | 2.54(1.91-3.33) | 2391(1802-3182) | 62.97(47.16-84.00) |
| Solomon Islands | 11(7-15) | 3.34(2.41-4.63) | 10(7-15) | 3.48(2.57-4.78) | 333(210-491) | 89.61(61.13-127.89) |
| Somalia | 838(515-1202) | 12.37(7.94-17.53) | 861(547-1230) | 13.21(8.48-18.69) | 25982(16246-36969) | 339.06(215.98-482.02) |
| South Africa | 4364(3860-5453) | 9.91(8.78-12.09) | 4508(4008-5540) | 10.57(9.46-12.54) | 114559(100475-146973) | 243.71(214.98-308.82) |
| South Sudan | 387(252-577) | 10.07(6.70-14.51) | 399(260-592) | 10.79(7.13-15.50) | 11159(7196-17375) | 259.65(169.11-388.52) |
| Spain | 2656(2048-3391) | 3.03(2.31-3.90) | 2330(2105-2571) | 2.55(2.32-2.81) | 50953(46227-56096) | 62.34(56.53-68.84) |
| Sri Lanka | 1170(853-1570) | 4.61(3.40-6.19) | 1115(823-1502) | 4.49(3.32-6.01) | 26741(19249-36195) | 102.04(73.71-138.42) |
| Sudan | 854(264-1242) | 4.74(1.48-6.85) | 872(270-1268) | 5.04(1.58-7.21) | 22589(6902-33197) | 113.57(34.85-166.03) |
| Suriname | 8(7-10) | 1.38(1.10-1.68) | 9(7-11) | 1.44(1.16-1.75) | 220(173-270) | 34.95(27.53-42.74) |
| Sweden | 608(505-725) | 2.95(2.45-3.52) | 581(530-628) | 2.71(2.50-2.92) | 10923(10100-11776) | 57.63(53.53-61.91) |
| Switzerland | 686(530-885) | 4.09(3.13-5.31) | 577(515-642) | 3.31(2.98-3.68) | 11576(10388-12867) | 73.50(65.93-81.93) |
| Syrian Arab Republic | 112(82-146) | 0.92(0.69-1.19) | 110(81-144) | 0.96(0.73-1.23) | 2883(2098-3836) | 21.75(16.07-28.46) |
| Taiwan  (Province of China) | 3864(2922-5131) | 9.99(7.58-13.27) | 3086(2342-4087) | 7.89(6.02-10.38) | 87728(66812-117387) | 230.22(176.24-308.61) |
| Tajikistan | 338(244-709) | 7.16(5.26-15.41) | 342(249-722) | 7.72(5.69-16.63) | 9645(6940-19644) | 175.32(127.84-369.48) |
| Thailand | 3246(2069-4396) | 3.13(2.01-4.22) | 3096(2001-4168) | 3.00(1.95-4.03) | 80034(49925-110058) | 76.41(48.12-104.93) |
| Timor-Leste | 19(13-27) | 2.32(1.69-3.40) | 19(14-28) | 2.48(1.82-3.60) | 486(337-711) | 57.30(40.51-84.21) |
| Togo | 171(95-237) | 4.66(2.56-6.32) | 173(96-238) | 4.92(2.69-6.63) | 4883(2700-6885) | 119.04(65.92-164.49) |
| Tokelau | 0(0-0) | 1.90(1.44-2.44) | 0(0-0) | 2.02(1.54-2.57) | 1(0-1) | 44.75(33.37-58.95) |
| Tonga | 2(1-2) | 2.07(1.57-2.67) | 2(1-2) | 2.21(1.67-2.86) | 40(29-52) | 49.84(36.94-65.43) |
| Trinidad and Tobago | 28(21-38) | 1.53(1.12-2.01) | 29(22-38) | 1.58(1.17-2.07) | 718(520-968) | 38.69(28.18-51.55) |
| Tunisia | 119(83-162) | 0.96(0.67-1.30) | 119(83-161) | 0.97(0.68-1.31) | 2767(1883-3781) | 21.41(14.66-29.20) |
| Turkey | 1380(1071-1734) | 1.56(1.21-1.96) | 1333(1032-1682) | 1.52(1.18-1.93) | 33554(25496-42068) | 36.71(28.11-46.04) |
| Turkmenistan | 376(294-470) | 9.58(7.60-11.95) | 379(298-472) | 9.97(7.94-12.44) | 10410(8081-13104) | 245.69(193.26-306.68) |
| Tuvalu | 0(0-0) | 2.30(1.68-3.10) | 0(0-0) | 2.45(1.80-3.30) | 6(4-8) | 57.20(41.52-77.97) |
| Uganda | 2203(1674-2805) | 15.61(12.06-19.47) | 2245(1719-2837) | 16.53(12.84-20.60) | 63186(47116-82060) | 403.54(306.25-514.39) |
| Ukraine | 2163(1724-2696) | 2.99(2.39-3.72) | 2033(1612-2494) | 2.78(2.20-3.42) | 57657(45456-71622) | 82.28(64.68-102.43) |
| United Arab Emirates | 383(114-655) | 8.08(2.54-13.84) | 355(104-607) | 8.42(2.68-14.24) | 13029(3794-22616) | 195.31(60.07-332.74) |
| United Kingdom | 10341(8560-12395) | 8.23(6.80-9.88) | 10037(9366-10481) | 7.77(7.31-8.09) | 188989(179710-195641) | 162.23(155.34-167.60) |
| United Republic of Tanzania | 2910(1881-4150) | 11.85(7.69-16.53) | 2986(1941-4214) | 12.56(8.13-17.39) | 81894(51622-119615) | 305.46(195.67-436.56) |
| United States of America | 23150(19583-27406) | 4.20(3.54-4.98) | 21615(20554-22521) | 3.86(3.69-4.02) | 471890(453307-489063) | 89.14(85.88-92.25) |
| United States Virgin Islands | 8(6-9) | 4.19(3.37-4.94) | 8(6-9) | 4.27(3.48-5.03) | 184(147-220) | 102.40(81.31-123.62) |
| Uruguay | 329(256-419) | 6.12(4.76-7.83) | 344(311-384) | 6.22(5.63-6.93) | 6876(6203-7622) | 136.92(123.14-152.13) |
| Uzbekistan | 1328(1088-1575) | 6.58(5.51-7.66) | 1320(1084-1562) | 6.98(5.88-8.09) | 38097(31208-45398) | 162.45(134.23-191.13) |
| Vanuatu | 4(3-6) | 2.56(1.87-3.55) | 4(3-6) | 2.75(2.02-3.80) | 120(85-171) | 64.80(46.37-90.68) |
| Venezuela  (Bolivarian Republic of) | 571(413-752) | 1.97(1.44-2.59) | 585(426-766) | 2.05(1.50-2.67) | 13890(9937-18605) | 46.49(33.57-62.02) |
| Viet Nam | 2824(1926-3620) | 2.89(2.04-3.65) | 2727(1899-3470) | 2.87(2.07-3.58) | 75348(49100-98541) | 72.98(49.39-93.95) |
| Yemen | 541(165-798) | 4.18(1.28-6.31) | 551(168-815) | 4.45(1.36-6.78) | 14931(4511-22259) | 103.13(31.29-151.92) |
| Zambia | 966(624-1329) | 14.10(9.26-18.84) | 975(635-1330) | 14.83(9.78-19.76) | 28731(18576-39831) | 374.27(242.43-512.78) |
| Zimbabwe | 1084(843-1346) | 15.40(12.22-18.95) | 1087(853-1348) | 16.02(12.75-19.64) | 31029(23759-39018) | 400.70(308.71-500.53) |

Abbreviations: ASIR, age-standardized incidence rate; ASDR, age-standardized mortality rate; DALYs, disability-adjusted life-years; UI, uncertain interval.
