# Supplementary material for: Burden of esophageal cancer and its attributable risk factors in 204 countries and territories from 1990 to 2019
Source: Front Public Health. 2022 Sep 6;10:952087. doi: 10.3389/fpubh.2022.952087 (PMC9485842; doi:10.3389/fpubh.2022.952087)
Supplement: Supplementary file 2 [file Table_2.docx]

**Supplementary Table 2. The trends in incidence, death and disability-adjusted life-years of esophageal cancer between 1990 and 2019.**

| **Characteristics** | **Relative change in incidence (95% UI)** | **EAPC of ASIR (95% CI)** | **Relative change in deaths (95% UI)** | **EAPC of ASMR (95% CI)** | **Relative change in DALY (95% UI)** | **EAPC of age-standardized DALY rate (95% CI)** |
| --- | --- | --- | --- | --- | --- | --- |
| Afghanistan | 59.69%(14.80%-119.35%) | -0.40(-0.52--0.27) | 57.42%(14.64%-114.82%) | -0.35(-0.47--0.23) | 70.82%(22.70%-139.56%) | -0.55(-0.70--0.39) |
| Albania | 37.46%(-0.34%-84.18%) | -1.08(-1.43--0.73) | 38.98%(1.73%-85.61%) | -1.14(-1.50--0.80) | 19.12%(-14.81%-62.03%) | -1.22(-1.56--0.89) |
| Algeria | 133.07%(63.13%-221.48%) | -0.77(-0.93--0.61) | 130.23%(61.65%-215.95%) | -0.84(-0.99--0.69) | 115.79%(50.02%-201.02%) | -0.97(-1.11--0.82) |
| American Samoa | 143.73%(82.58%-209.27%) | 0.54(0.36-0.72) | 145.55%(84.76%-209.60%) | 0.47(0.29-0.65) | 127.09%(67.56%-194.84%) | 0.50(0.32-0.68) |
| Andorra | 135.89%(56.41%-239.74%) | -0.34(-0.42--0.26) | 119.36%(46.73%-215.43%) | -0.71(-0.76--0.65) | 103.39%(34.77%-195.99%) | -0.75(-0.81--0.68) |
| Angola | 103.88%(41.60%-206.61%) | -1.34(-1.46--1.22) | 103.24%(42.33%-202.29%) | -1.31(-1.42--1.19) | 95.00%(32.80%-196.06%) | -1.57(-1.69--1.44) |
| Antigua and Barbuda | 76.41%(45.17%-114.26%) | -0.29(-0.55--0.03) | 68.82%(39.22%-103.32%) | -0.30(-0.57--0.04) | 80.37%(47.50%-121.19%) | -0.52(-0.78--0.25) |
| Argentina | 16.13%(-8.74%-45.78%) | -1.60(-1.76--1.44) | 14.02%(4.10%-25.20%) | -1.70(-1.85--1.55) | 4.82%(-4.13%-14.94%) | -1.82(-1.97--1.67) |
| Armenia | 3.54%(-15.64%-24.75%) | -1.32(-1.63--1.02) | 5.96%(-13.61%-27.32%) | -1.32(-1.62--1.02) | -7.16%(-24.83%-13.31%) | -1.50(-1.82--1.18) |
| Australia | 105.44%(59.97%-163.92%) | -0.30(-0.42--0.18) | 105.96%(86.36%-127.72%) | -0.34(-0.43--0.25) | 83.40%(66.42%-102.17%) | -0.44(-0.52--0.37) |
| Austria | 28.13%(2.61%-59.71%) | -0.75(-0.89--0.61) | 15.15%(4.08%-26.33%) | -1.11(-1.22--1.02) | 6.56%(-4.01%-17.39%) | -1.34(-1.46--1.23) |
| Azerbaijan | 58.79%(20.09%-129.62%) | -0.44(-0.63--0.24) | 59.00%(19.69%-129.85%) | -0.29(-0.47--0.10) | 52.54%(15.02%-120.74%) | -0.87(-1.07--0.66) |
| Bahamas | 101.75%(54.82%-161.82%) | -0.61(-0.80--0.42) | 100.77%(54.25%-158.50%) | -0.61(-0.81--0.41) | 96.58%(49.81%-158.14%) | -0.72(-0.92--0.50) |
| Bahrain | 154.09%(73.21%-350.19%) | -3.36(-3.70--3.03) | 137.76%(61.93%-322.31%) | -3.43(-3.76--3.10) | 148.51%(68.17%-338.03%) | -3.82(-4.16--3.47) |
| Bangladesh | 75.56%(16.13%-181.14%) | -1.46(-1.60--1.32) | 77.09%(17.11%-181.22%) | -1.46(-1.60--1.31) | 63.10%(5.65%-167.26%) | -1.59(-1.72--1.45) |
| Barbados | 51.65%(20.66%-83.91%) | -0.72(-0.86--0.59) | 47.38%(17.94%-77.94%) | -0.78(-0.91--0.63) | 51.37%(19.16%-86.03%) | -0.84(-0.96--0.70) |
| Belarus | 20.31%(-9.26%-59.44%) | -0.57(-0.75--0.37) | 16.85%(-11.35%-54.31%) | -0.69(-0.88--0.50) | 14.58%(-15.05%-54.18%) | -0.73(-0.93--0.52) |
| Belgium | 87.48%(45.26%-138.27%) | 0.91(0.70-1.12) | 71.30%(55.87%-88.33%) | 0.50(0.33-0.67) | 57.87%(43.99%-72.84%) | 0.32(0.10-0.55) |
| Belize | 275.00%(206.84%-353.77%) | 0.56(0.18-0.93) | 259.07%(197.96%-328.66%) | 0.47(0.09-0.85) | 306.05%(232.23%-389.02%) | 0.64(0.27-1.02) |
| Benin | 227.36%(128.40%-341.27%) | 1.49(1.29-1.69) | 222.54%(126.06%-331.87%) | 1.49(1.29-1.69) | 235.35%(130.60%-359.16%) | 1.39(1.20-1.58) |
| Bermuda | 30.55%(4.28%-61.75%) | -1.38(-1.65--1.11) | 25.22%(0.22%-54.57%) | -1.64(-1.91--1.37) | 9.69%(-12.58%-38.15%) | -1.72(-2.03--1.42) |
| Bhutan | 91.70%(26.73%-200.28%) | -0.46(-0.61--0.31) | 97.69%(32.08%-204.86%) | -0.41(-0.55--0.27) | 67.64%(8.75%-173.27%) | -0.78(-0.93--0.63) |
| Bolivia  (Plurinational State of) | 149.34%(78.77%-258.42%) | -0.41(-0.53--0.30) | 153.91%(82.93%-260.14%) | -0.35(-0.46--0.24) | 126.65%(59.84%-238.14%) | -0.69(-0.80--0.57) |
| Bosnia and Herzegovina | 18.06%(-9.06%-52.04%) | -0.99(-1.12--0.86) | 18.76%(-8.65%-52.43%) | -1.11(-1.24--0.98) | 7.20%(-18.65%-38.70%) | -1.02(-1.16--0.88) |
| Botswana | 123.21%(48.56%-231.23%) | -0.93(-1.39--0.46) | 118.46%(46.27%-222.87%) | -0.98(-1.45--0.51) | 124.00%(42.82%-242.87%) | -1.11(-1.65--0.58) |
| Brazil | 105.73%(92.64%-118.52%) | -0.87(-0.92--0.81) | 104.61%(92.08%-117.94%) | -0.95(-0.99--0.90) | 91.97%(80.23%-104.07%) | -0.96(-1.01--0.89) |
| Brunei Darussalam | 98.73%(54.39%-192.78%) | -1.67(-1.90--1.44) | 79.93%(40.88%-164.69%) | -1.93(-2.15--1.72) | 79.77%(38.12%-165.66%) | -2.08(-2.35--1.81) |
| Bulgaria | -14.88%(-34.43%-8.05%) | -0.13(-0.80-0.55) | -15.36%(-34.68%-6.99%) | -0.27(-0.92-0.38) | -19.97%(-38.50%-2.38%) | -0.12(-0.81-0.58) |
| Burkina Faso | 175.54%(94.61%-265.26%) | 1.32(1.13-1.52) | 173.65%(93.10%-262.29%) | 1.33(1.14-1.52) | 180.78%(98.36%-274.94%) | 1.28(1.08-1.47) |
| Burundi | 34.54%(-9.77%-116.79%) | -1.76(-1.93--1.58) | 32.90%(-9.57%-111.04%) | -1.72(-1.90--1.56) | 36.43%(-10.57%-128.11%) | -1.92(-2.11--1.74) |
| Cabo Verde | 211.56%(152.02%-278.84%) | 0.92(0.52-1.32) | 200.65%(143.64%-265.92%) | 0.82(0.40-1.25) | 237.19%(166.51%-316.91%) | 1.03(0.67-1.38) |
| Cambodia | 107.79%(42.22%-212.90%) | -0.89(-1.04--0.72) | 108.33%(42.16%-210.71%) | -0.88(-1.03--0.73) | 91.40%(28.49%-198.75%) | -1.13(-1.28--0.98) |
| Cameroon | 257.44%(123.92%-413.92%) | 1.35(1.16-1.53) | 254.32%(124.17%-410.64%) | 1.33(1.15-1.51) | 257.23%(124.34%-419.65%) | 1.28(1.09-1.46) |
| Canada | 146.73%(90.92%-210.84%) | 0.53(0.44-0.63) | 125.23%(103.64%-149.90%) | 0.11(0.02-0.19) | 107.52%(87.34%-129.84%) | 0.03(-0.05-0.11) |
| Central African Republic | 42.43%(5.24%-98.42%) | -1.00(-1.09--0.91) | 41.09%(4.82%-96.13%) | -1.00(-1.09--0.91) | 43.93%(5.02%-103.45%) | -1.09(-1.19--0.99) |
| Chad | 193.24%(108.05%-284.29%) | 1.84(1.64-2.03) | 188.96%(104.47%-276.60%) | 1.85(1.65-2.05) | 203.14%(114.92%-303.80%) | 1.78(1.57-1.97) |
| Chile | 31.46%(3.73%-65.14%) | -2.82(-3.06--2.58) | 29.23%(14.54%-44.54%) | -3.02(-3.27--2.78) | 9.54%(-2.13%-22.79%) | -3.36(-3.60--3.12) |
| China | 60.13%(28.11%-114.00%) | -1.58(-2.05--1.11) | 45.70%(16.47%-100.59%) | -1.96(-2.44--1.47) | 28.17%(0.11%-84.53%) | -2.27(-2.78--1.75) |
| Colombia | 47.98%(12.59%-90.11%) | -3.11(-3.33--2.89) | 48.32%(13.35%-89.26%) | -3.23(-3.44--3.01) | 27.00%(-4.69%-64.69%) | -3.32(-3.55--3.10) |
| Comoros | 72.98%(18.59%-231.51%) | -1.13(-1.33--0.95) | 73.14%(20.15%-219.63%) | -1.12(-1.31--0.94) | 69.87%(11.85%-256.77%) | -1.25(-1.47--1.03) |
| Congo | 63.98%(16.52%-132.21%) | -1.58(-1.76--1.39) | 63.02%(16.77%-127.97%) | -1.54(-1.72--1.36) | 60.91%(10.28%-135.28%) | -1.82(-2.02--1.62) |
| Cook Islands | 64.99%(26.23%-108.85%) | -0.91(-1.03--0.78) | 62.30%(24.74%-104.81%) | -1.07(-1.20--0.94) | 46.02%(9.91%-89.20%) | -1.03(-1.13--0.93) |
| Costa Rica | 88.36%(43.42%-145.18%) | -1.99(-2.25--1.73) | 86.50%(42.54%-141.75%) | -2.07(-2.32--1.82) | 71.69%(29.39%-124.75%) | -2.24(-2.51--1.97) |
| Croatia | -8.48%(-30.46%-19.59%) | -1.11(-1.25--0.98) | -10.25%(-32.26%-17.04%) | -1.23(-1.37--1.10) | -22.64%(-41.50%-2.57%) | -1.52(-1.66--1.37) |
| Cuba | 161.46%(106.17%-223.38%) | 1.66(1.45-1.88) | 147.64%(96.64%-205.03%) | 1.41(1.20-1.63) | 165.50%(108.00%-231.03%) | 1.82(1.61-2.02) |
| Cyprus | 180.24%(78.33%-278.23%) | 0.71(0.39-1.05) | 145.40%(53.52%-228.21%) | 0.13(-0.17-0.43) | 122.97%(44.59%-195.64%) | -0.01(-0.29-0.28) |
| Czechia | 72.49%(37.47%-119.42%) | 0.59(0.45-0.73) | 61.15%(28.42%-102.73%) | 0.30(0.19-0.41) | 48.55%(17.68%-89.92%) | 0.13(-0.02-0.27) |
| Côte d'Ivoire | 234.40%(130.74%-360.05%) | 1.05(0.90-1.18) | 235.78%(131.77%-359.01%) | 1.07(0.92-1.20) | 224.47%(121.55%-356.04%) | 0.92(0.78-1.06) |
| Democratic People's Republic of Korea | 75.00%(31.51%-141.76%) | -0.44(-0.51--0.37) | 75.89%(33.04%-143.27%) | -0.51(-0.59--0.43) | 61.90%(18.72%-129.58%) | -0.53(-0.61--0.46) |
| Democratic Republic of the Congo | 77.74%(29.62%-147.07%) | -0.99(-1.07--0.90) | 77.97%(31.14%-145.61%) | -1.00(-1.08--0.91) | 76.54%(25.84%-150.84%) | -1.04(-1.13--0.96) |
| Denmark | 44.35%(11.95%-84.97%) | 0.01(-0.14-0.16) | 32.02%(17.63%-48.68%) | -0.44(-0.57--0.31) | 20.36%(7.22%-35.69%) | -0.76(-0.88--0.63) |
| Djibouti | 265.63%(147.78%-450.84%) | -0.70(-0.83--0.56) | 269.90%(151.76%-455.47%) | -0.67(-0.79--0.54) | 255.46%(135.92%-449.19%) | -0.76(-0.90--0.61) |
| Dominica | 25.58%(-0.72%-60.15%) | 0.09(-0.09-0.27) | 24.08%(-1.68%-57.99%) | 0.09(-0.09-0.28) | 30.18%(1.47%-68.66%) | 0.05(-0.14-0.23) |
| Dominican Republic | 273.59%(146.63%-423.77%) | 1.92(1.72-2.12) | 271.96%(148.97%-422.15%) | 1.87(1.67-2.06) | 254.69%(130.16%-410.14%) | 1.85(1.65-2.05) |
| Ecuador | 110.26%(56.95%-209.22%) | -0.89(-1.11--0.66) | 111.51%(58.66%-207.23%) | -0.87(-1.09--0.64) | 88.90%(39.68%-186.84%) | -1.20(-1.44--0.98) |
| Egypt | 167.70%(84.69%-267.00%) | 0.82(0.70-0.94) | 162.85%(81.79%-260.21%) | 0.77(0.65-0.90) | 155.69%(75.56%-251.86%) | 0.68(0.58-0.79) |
| El Salvador | 132.21%(73.80%-204.11%) | 0.03(-0.36-0.43) | 135.96%(76.78%-207.47%) | -0.01(-0.41-0.39) | 105.27%(51.08%-175.27%) | -0.19(-0.56-0.18) |
| Equatorial Guinea | 64.37%(-4.98%-205.05%) | -1.25(-1.52--0.99) | 65.64%(-2.79%-204.83%) | -1.15(-1.41--0.90) | 51.82%(-14.22%-188.55%) | -1.72(-2.02--1.40) |
| Eritrea | 129.98%(60.35%-242.05%) | -0.62(-0.84--0.39) | 130.47%(60.95%-240.41%) | -0.61(-0.84--0.38) | 119.33%(51.32%-229.73%) | -0.82(-1.04--0.60) |
| Estonia | 9.15%(-18.58%-41.57%) | -0.18(-0.45-0.09) | 8.40%(-18.93%-40.03%) | -0.29(-0.56--0.03) | -5.22%(-30.55%-23.84%) | -0.60(-0.85--0.34) |
| Eswatini | 89.48%(30.52%-175.29%) | -0.34(-0.96-0.29) | 88.69%(32.67%-171.15%) | -0.34(-0.97-0.30) | 90.84%(27.46%-186.38%) | -0.29(-0.99-0.41) |
| Ethiopia | 17.77%(-22.29%-110.32%) | -1.98(-2.12--1.84) | 21.54%(-20.17%-112.34%) | -1.88(-2.01--1.74) | 7.98%(-30.76%-100.90%) | -2.32(-2.47--2.18) |
| Fiji | 131.49%(55.63%-225.82%) | 0.73(0.45-1.01) | 132.43%(56.74%-225.87%) | 0.72(0.44-1.00) | 117.13%(44.19%-207.98%) | 0.66(0.38-0.94) |
| Finland | 57.97%(20.31%-101.60%) | 0.08(-0.03-0.18) | 43.56%(27.95%-60.95%) | -0.42(-0.54--0.30) | 32.71%(17.06%-49.68%) | -0.36(-0.47--0.26) |
| France | -4.16%(-25.26%-22.36%) | -2.03(-2.11--1.94) | -11.47%(-19.79%--2.35%) | -2.44(-2.54--2.34) | -25.72%(-32.90%--18.14%) | -2.85(-2.97--2.73) |
| Gabon | 57.26%(11.51%-116.41%) | -0.80(-0.93--0.67) | 55.78%(11.29%-113.16%) | -0.80(-0.93--0.66) | 56.46%(8.32%-119.96%) | -0.96(-1.10--0.81) |
| Gambia | 251.63%(145.18%-416.78%) | 0.79(0.62-0.97) | 254.50%(147.27%-412.95%) | 0.80(0.63-0.97) | 242.82%(132.82%-409.86%) | 0.77(0.58-0.97) |
| Georgia | -15.16%(-32.51%-4.37%) | 0.58(-0.03-1.21) | -12.49%(-30.13%-7.44%) | 0.62(0.01-1.25) | -21.38%(-37.67%--2.35%) | 0.49(-0.11-1.11) |
| Germany | 119.41%(69.40%-186.60%) | 1.33(0.98-1.67) | 81.05%(65.45%-99.35%) | 0.61(0.37-0.86) | 52.49%(38.50%-68.14%) | 0.17(-0.04-0.37) |
| Ghana | 173.21%(97.53%-268.86%) | 0.44(0.30-0.57) | 173.06%(99.13%-266.86%) | 0.45(0.31-0.58) | 164.81%(87.61%-263.05%) | 0.35(0.21-0.48) |
| Greece | 3.10%(-19.82%-30.76%) | -1.64(-1.75--1.53) | -0.88%(-10.14%-9.66%) | -2.01(-2.14--1.88) | -9.60%(-17.89%-0.13%) | -1.64(-1.73--1.54) |
| Greenland | 76.11%(37.72%-118.41%) | -0.69(-0.86--0.51) | 75.77%(36.34%-119.09%) | -0.75(-0.92--0.57) | 54.55%(18.62%-98.10%) | -0.91(-1.04--0.77) |
| Grenada | 49.93%(25.65%-75.38%) | -0.17(-0.29--0.05) | 41.65%(19.18%-65.36%) | -0.24(-0.37--0.12) | 60.46%(34.34%-90.89%) | -0.19(-0.32--0.05) |
| Guam | 166.87%(102.90%-246.73%) | 0.26(-0.21-0.74) | 170.64%(106.78%-250.94%) | 0.04(-0.48-0.55) | 149.72%(87.82%-226.46%) | 0.61(0.11-1.11) |
| Guatemala | 290.78%(195.32%-421.07%) | 0.46(-0.07-1.00) | 299.18%(207.75%-433.04%) | 0.39(-0.17-0.95) | 255.09%(169.42%-379.22%) | 0.43(-0.08-0.94) |
| Guinea | 91.83%(40.33%-155.75%) | 1.06(0.86-1.26) | 90.22%(40.74%-152.87%) | 1.08(0.87-1.27) | 95.68%(42.63%-167.48%) | 0.96(0.77-1.17) |
| Guinea-Bissau | 107.69%(39.37%-191.67%) | 0.76(0.66-0.87) | 106.31%(38.69%-189.41%) | 0.74(0.64-0.86) | 109.47%(40.41%-199.95%) | 0.78(0.67-0.90) |
| Guyana | 58.69%(13.53%-112.93%) | -0.19(-0.32--0.05) | 56.57%(13.01%-109.67%) | -0.23(-0.36--0.10) | 60.97%(12.77%-117.92%) | -0.09(-0.22-0.04) |
| Haiti | 68.38%(23.62%-137.05%) | -0.63(-0.81--0.44) | 68.93%(25.20%-135.42%) | -0.62(-0.80--0.44) | 65.79%(18.57%-137.52%) | -0.71(-0.91--0.50) |
| Honduras | 309.59%(204.43%-442.08%) | 1.55(1.36-1.74) | 313.78%(207.51%-445.36%) | 1.62(1.42-1.82) | 277.12%(179.22%-405.93%) | 1.29(1.11-1.46) |
| Hungary | 10.21%(-11.37%-36.66%) | -1.00(-1.42--0.58) | 10.72%(-10.69%-36.67%) | -1.00(-1.41--0.59) | -3.57%(-22.85%-20.54%) | -1.48(-1.91--1.04) |
| Iceland | 89.68%(55.55%-129.17%) | -0.20(-0.29--0.11) | 70.73%(45.62%-98.91%) | -0.59(-0.70--0.47) | 65.03%(40.05%-92.08%) | -0.60(-0.74--0.45) |
| India | 110.88%(72.32%-154.91%) | -0.94(-1.10--0.77) | 112.75%(74.67%-158.27%) | -1.00(-1.18--0.84) | 96.81%(60.79%-139.39%) | -0.93(-1.08--0.77) |
| Indonesia | 103.68%(57.00%-168.82%) | -0.10(-0.15--0.06) | 104.96%(58.47%-168.74%) | -0.07(-0.12--0.03) | 88.20%(42.92%-148.83%) | -0.36(-0.39--0.32) |
| Iran  (Islamic Republic of) | 126.45%(87.23%-180.25%) | -0.86(-0.96--0.76) | 126.43%(83.92%-178.17%) | -0.95(-1.03--0.86) | 93.45%(59.52%-140.58%) | -1.20(-1.30--1.10) |
| Iraq | 200.78%(116.85%-323.66%) | -0.15(-0.23--0.08) | 189.28%(110.17%-304.47%) | -0.21(-0.29--0.14) | 199.88%(111.98%-330.07%) | -0.30(-0.36--0.24) |
| Ireland | 87.41%(43.56%-145.89%) | -0.05(-0.14-0.04) | 61.88%(39.77%-86.09%) | -0.68(-0.76--0.58) | 53.67%(32.66%-75.83%) | -0.80(-0.89--0.70) |
| Israel | 105.71%(55.99%-165.72%) | -1.04(-1.19--0.91) | 86.19%(62.36%-110.02%) | -1.47(-1.59--1.34) | 76.16%(54.83%-97.34%) | -1.42(-1.57--1.27) |
| Italy | -0.90%(-18.99%-18.72%) | -1.77(-1.86--1.68) | -5.62%(-12.82%-0.23%) | -2.10(-2.21--1.99) | -20.87%(-25.57%--16.28%) | -2.33(-2.46--2.20) |
| Jamaica | 46.16%(9.99%-90.27%) | -0.56(-0.91--0.21) | 43.71%(8.67%-88.05%) | -0.61(-0.95--0.26) | 49.50%(11.25%-97.30%) | -0.60(-0.98--0.23) |
| Japan | 90.77%(58.82%-126.89%) | -0.09(-0.30-0.13) | 67.22%(54.26%-77.24%) | -0.88(-0.99--0.76) | 31.18%(23.30%-38.84%) | -1.08(-1.26--0.91) |
| Jordan | 330.29%(217.37%-473.46%) | -0.77(-0.88--0.65) | 310.96%(205.01%-442.03%) | -0.93(-1.04--0.80) | 303.32%(195.45%-437.73%) | -1.00(-1.13--0.88) |
| Kazakhstan | -49.93%(-57.24%--41.39%) | -3.60(-3.77--3.43) | -50.42%(-57.61%--42.17%) | -3.61(-3.78--3.45) | -51.49%(-58.68%--42.96%) | -3.75(-3.92--3.59) |
| Kenya | 218.84%(155.49%-281.36%) | 0.90(0.69-1.13) | 243.16%(177.40%-315.79%) | 1.38(1.12-1.64) | 246.51%(179.63%-323.05%) | 1.21(0.93-1.48) |
| Kiribati | 74.10%(24.70%-134.52%) | -0.29(-0.44--0.15) | 70.50%(22.31%-129.66%) | -0.29(-0.42--0.15) | 73.92%(24.76%-137.81%) | -0.44(-0.58--0.29) |
| Kuwait | 234.63%(167.96%-319.10%) | -0.70(-1.05--0.35) | 231.45%(167.86%-310.15%) | -0.77(-1.13--0.41) | 183.11%(121.45%-257.77%) | -1.29(-1.66--0.93) |
| Kyrgyzstan | -26.06%(-38.04%--12.66%) | -2.62(-2.79--2.43) | -25.12%(-37.12%--11.90%) | -2.47(-2.63--2.31) | -29.01%(-40.65%--15.71%) | -2.98(-3.20--2.76) |
| Lao People's Democratic Republic | 25.63%(-14.05%-91.84%) | -1.94(-2.09--1.80) | 25.71%(-13.29%-89.60%) | -1.91(-2.06--1.77) | 19.74%(-19.73%-88.00%) | -2.21(-2.35--2.06) |
| Latvia | 13.04%(-11.54%-44.58%) | 0.55(0.25-0.85) | 13.70%(-10.74%-45.26%) | 0.51(0.21-0.81) | 4.07%(-19.75%-34.24%) | 0.34(0.04-0.65) |
| Lebanon | 133.68%(66.77%-249.35%) | 0.22(0.01-0.42) | 119.86%(58.05%-227.99%) | -0.12(-0.28-0.04) | 92.17%(37.54%-184.57%) | -0.27(-0.48--0.06) |
| Lesotho | 59.59%(7.69%-125.40%) | 1.01(0.77-1.23) | 58.81%(9.10%-121.97%) | 1.01(0.76-1.24) | 67.23%(8.46%-141.85%) | 1.12(0.84-1.39) |
| Liberia | 134.93%(53.67%-236.37%) | 1.49(1.15-1.84) | 130.73%(51.79%-225.76%) | 1.50(1.16-1.85) | 148.03%(61.42%-260.68%) | 1.39(1.03-1.74) |
| Libya | 214.78%(123.12%-346.62%) | 0.50(0.38-0.63) | 207.74%(120.16%-334.39%) | 0.43(0.31-0.56) | 212.93%(118.12%-347.17%) | 0.38(0.26-0.51) |
| Lithuania | 43.08%(13.14%-78.36%) | 1.05(0.74-1.34) | 42.08%(13.01%-76.43%) | 0.94(0.66-1.24) | 30.86%(2.55%-65.92%) | 0.84(0.52-1.18) |
| Luxembourg | 40.53%(11.00%-78.09%) | -1.00(-1.18--0.82) | 28.65%(8.22%-53.93%) | -1.37(-1.49--1.25) | 18.56%(-1.33%-44.13%) | -1.62(-1.74--1.49) |
| Madagascar | 75.90%(26.14%-148.35%) | -0.92(-1.08--0.74) | 73.60%(25.33%-142.57%) | -0.91(-1.08--0.73) | 79.97%(28.41%-156.05%) | -1.00(-1.16--0.84) |
| Malawi | 110.37%(55.82%-182.74%) | 0.49(0.15-0.84) | 111.64%(58.64%-182.44%) | 0.49(0.17-0.83) | 107.00%(49.64%-184.90%) | 0.42(0.06-0.78) |
| Malaysia | 181.51%(111.25%-272.33%) | -0.57(-0.76--0.37) | 170.23%(104.53%-256.32%) | -0.71(-0.91--0.51) | 152.75%(85.79%-236.27%) | -0.87(-1.07--0.66) |
| Maldives | 62.32%(2.91%-240.98%) | -2.92(-3.19--2.63) | 54.62%(-1.04%-218.30%) | -3.14(-3.39--2.88) | 28.20%(-19.98%-179.54%) | -3.77(-4.06--3.47) |
| Mali | 107.67%(53.53%-183.98%) | 0.05(-0.01-0.10) | 107.80%(54.94%-179.93%) | 0.06(0.01-0.11) | 105.29%(48.42%-186.63%) | -0.04(-0.11-0.02) |
| Malta | 80.28%(42.89%-129.25%) | -0.53(-0.60--0.45) | 61.69%(32.19%-95.76%) | -1.00(-1.07--0.94) | 45.41%(20.19%-75.59%) | -0.92(-0.97--0.86) |
| Marshall Islands | 89.85%(40.00%-155.87%) | -0.35(-0.43--0.27) | 83.81%(35.14%-147.44%) | -0.36(-0.44--0.27) | 98.27%(45.29%-167.29%) | -0.40(-0.48--0.33) |
| Mauritania | 98.74%(30.14%-184.51%) | 0.41(0.17-0.65) | 101.12%(33.20%-183.95%) | 0.47(0.24-0.71) | 87.91%(20.26%-177.91%) | 0.16(-0.08-0.40) |
| Mauritius | 88.95%(50.23%-138.52%) | -1.05(-1.20--0.90) | 84.32%(47.06%-131.90%) | -1.17(-1.32--1.04) | 71.00%(34.75%-116.98%) | -1.21(-1.36--1.06) |
| Mexico | 112.37%(79.62%-149.40%) | -1.14(-1.29--1.00) | 107.35%(75.13%-144.37%) | -1.30(-1.45--1.15) | 103.15%(70.43%-141.45%) | -1.10(-1.24--0.95) |
| Micronesia  (Federated States of) | 37.65%(-8.50%-98.33%) | -0.52(-0.59--0.46) | 34.39%(-10.40%-92.82%) | -0.55(-0.61--0.48) | 37.28%(-10.76%-101.64%) | -0.62(-0.67--0.56) |
| Monaco | 55.27%(12.64%-109.81%) | 0.69(0.53-0.86) | 42.00%(5.12%-89.49%) | 0.29(0.14-0.43) | 36.11%(-1.74%-83.85%) | 0.16(0.05-0.28) |
| Mongolia | 78.17%(30.64%-142.98%) | -0.83(-1.09--0.57) | 74.52%(28.85%-136.53%) | -0.78(-1.04--0.52) | 86.78%(36.95%-157.26%) | -0.96(-1.21--0.70) |
| Montenegro | 77.52%(36.05%-127.04%) | 0.62(0.46-0.79) | 78.57%(36.49%-127.02%) | 0.60(0.44-0.77) | 63.25%(24.73%-110.24%) | 0.43(0.23-0.62) |
| Morocco | 150.11%(80.54%-239.57%) | 0.23(0.07-0.40) | 150.02%(82.96%-235.98%) | 0.24(0.07-0.42) | 139.73%(70.14%-229.33%) | 0.11(-0.03-0.24) |
| Mozambique | 131.91%(67.95%-225.85%) | 1.14(1.00-1.27) | 128.70%(65.86%-220.42%) | 1.07(0.94-1.19) | 136.26%(69.42%-239.89%) | 1.25(1.09-1.40) |
| Myanmar | 30.13%(-12.12%-96.74%) | -1.55(-1.61--1.49) | 30.69%(-8.87%-89.90%) | -1.54(-1.60--1.48) | 18.78%(-20.24%-80.02%) | -1.86(-1.93--1.79) |
| Namibia | 112.30%(56.65%-199.87%) | 0.06(-0.23-0.34) | 109.73%(56.55%-192.25%) | 0.02(-0.27-0.31) | 113.33%(52.70%-206.94%) | -0.04(-0.37-0.29) |
| Nauru | -1.71%(-27.97%-28.68%) | -0.73(-1.03--0.43) | -5.09%(-30.21%-23.60%) | -0.72(-1.01--0.42) | -0.21%(-26.68%-33.21%) | -0.88(-1.20--0.54) |
| Nepal | 117.39%(50.94%-200.33%) | -0.04(-0.42-0.35) | 122.34%(57.24%-207.50%) | 0.02(-0.36-0.40) | 94.45%(34.24%-177.50%) | -0.30(-0.70-0.10) |
| Netherlands | 215.48%(142.38%-299.33%) | 2.20(1.85-2.57) | 184.17%(157.37%-213.59%) | 1.75(1.40-2.08) | 162.90%(138.04%-189.50%) | 1.54(1.17-1.92) |
| New Zealand | 95.46%(57.35%-140.17%) | -0.23(-0.31--0.16) | 74.30%(55.22%-95.90%) | -0.72(-0.79--0.64) | 61.09%(43.93%-79.71%) | -0.83(-0.91--0.75) |
| Nicaragua | 201.84%(132.40%-304.36%) | -0.29(-0.54--0.05) | 200.38%(132.53%-301.36%) | -0.30(-0.58--0.02) | 172.77%(109.15%-267.20%) | -0.62(-0.84--0.40) |
| Niger | 218.64%(128.11%-323.94%) | 0.96(0.78-1.15) | 221.34%(130.15%-325.70%) | 1.01(0.82-1.20) | 205.45%(118.65%-314.22%) | 0.79(0.61-0.98) |
| Nigeria | 97.82%(38.95%-181.44%) | 0.33(0.16-0.50) | 99.21%(41.17%-193.11%) | 0.40(0.24-0.57) | 95.21%(35.25%-192.50%) | 0.08(-0.08-0.24) |
| Niue | -4.50%(-26.50%-21.17%) | -0.32(-0.46--0.18) | -10.11%(-30.85%-12.81%) | -0.45(-0.59--0.31) | -6.51%(-29.88%-19.97%) | -0.58(-0.73--0.43) |
| North Macedonia | 77.65%(35.72%-132.89%) | 0.09(-0.08-0.27) | 76.80%(35.12%-130.80%) | 0.06(-0.12-0.24) | 64.41%(25.59%-117.66%) | -0.09(-0.28-0.10) |
| Northern Mariana Islands | 469.97%(163.95%-691.28%) | 3.14(2.69-3.59) | 473.38%(164.88%-688.25%) | 3.10(2.64-3.55) | 385.45%(119.09%-598.04%) | 3.04(2.58-3.50) |
| Norway | 56.30%(30.00%-83.89%) | 0.51(0.36-0.66) | 41.44%(31.92%-53.60%) | 0.08(-0.01-0.17) | 34.79%(25.46%-47.33%) | -0.14(-0.24--0.04) |
| Oman | 148.25%(70.32%-245.97%) | 0.36(0.12-0.60) | 131.92%(61.52%-224.92%) | 0.26(0.05-0.47) | 131.58%(54.45%-231.68%) | -0.17(-0.50-0.16) |
| Pakistan | 122.55%(74.23%-184.77%) | 0.27(0.04-0.50) | 116.87%(70.76%-176.84%) | 0.25(0.03-0.48) | 131.81%(80.30%-197.95%) | 0.22(-0.03-0.47) |
| Palau | 132.03%(66.29%-217.06%) | 0.00(-0.13-0.12) | 121.03%(58.37%-198.95%) | -0.11(-0.23-0.00) | 129.28%(60.94%-218.46%) | -0.07(-0.19-0.05) |
| Palestine | 114.34%(53.10%-217.05%) | -1.02(-1.16--0.89) | 106.21%(48.88%-208.35%) | -1.09(-1.22--0.96) | 115.80%(52.56%-222.08%) | -1.16(-1.28--1.04) |
| Panama | 166.51%(97.76%-250.27%) | 0.15(-0.08-0.39) | 161.66%(95.22%-242.72%) | 0.05(-0.18-0.28) | 152.04%(85.10%-235.37%) | 0.12(-0.14-0.38) |
| Papua New Guinea | 153.10%(86.51%-239.55%) | -0.04(-0.10-0.01) | 154.00%(89.07%-239.41%) | -0.02(-0.07-0.04) | 154.06%(83.77%-246.71%) | -0.10(-0.16--0.03) |
| Paraguay | 210.48%(125.37%-325.46%) | 0.52(0.34-0.70) | 203.32%(121.86%-312.01%) | 0.44(0.25-0.63) | 204.60%(117.46%-324.53%) | 0.46(0.30-0.63) |
| Peru | 102.98%(47.32%-179.40%) | -1.00(-1.14--0.86) | 103.29%(47.71%-177.93%) | -1.07(-1.22--0.94) | 76.87%(24.99%-151.28%) | -1.28(-1.43--1.13) |
| Philippines | 121.12%(65.24%-209.41%) | -0.80(-0.98--0.61) | 118.93%(67.26%-198.26%) | -0.85(-1.04--0.67) | 112.51%(62.57%-191.40%) | -0.85(-1.03--0.67) |
| Poland | 38.97%(13.56%-69.38%) | -0.57(-0.66--0.47) | 38.86%(13.67%-69.23%) | -0.65(-0.73--0.56) | 29.92%(4.91%-60.73%) | -0.66(-0.77--0.55) |
| Portugal | 13.66%(-14.20%-48.01%) | -0.97(-1.12--0.80) | 5.52%(-5.06%-17.53%) | -1.37(-1.49--1.24) | 3.76%(-6.78%-15.63%) | -1.01(-1.22--0.81) |
| Puerto Rico | -23.42%(-42.21%--0.93%) | -3.62(-3.89--3.36) | -25.87%(-43.79%--3.90%) | -3.90(-4.18--3.62) | -31.94%(-48.83%--10.97%) | -3.75(-4.02--3.48) |
| Qatar | 502.38%(290.65%-879.63%) | 0.09(-0.20-0.38) | 444.33%(253.13%-787.41%) | 0.03(-0.27-0.32) | 449.76%(249.67%-797.33%) | -0.91(-1.20--0.60) |
| Republic of Korea | 91.67%(47.52%-278.93%) | -2.36(-2.66--2.07) | 33.43%(11.39%-169.61%) | -3.82(-4.15--3.48) | 2.51%(-16.12%-137.38%) | -4.34(-4.70--3.99) |
| Republic of Moldova | -7.70%(-22.19%-9.49%) | -0.93(-1.41--0.43) | -7.83%(-21.96%-8.96%) | -0.99(-1.47--0.50) | -12.65%(-26.56%-3.98%) | -1.00(-1.48--0.51) |
| Romania | 99.87%(59.41%-143.34%) | 1.53(1.26-1.81) | 96.67%(57.95%-139.92%) | 1.38(1.11-1.64) | 89.59%(49.89%-130.91%) | 1.51(1.20-1.82) |
| Russian Federation | -10.09%(-23.50%-4.72%) | -1.58(-1.82--1.32) | -13.67%(-26.47%-0.71%) | -1.79(-2.02--1.55) | -15.32%(-28.82%-0.03%) | -1.72(-1.97--1.45) |
| Rwanda | 19.93%(-18.87%-101.16%) | -2.64(-2.98--2.30) | 20.59%(-17.06%-99.96%) | -2.59(-2.93--2.25) | 15.34%(-24.89%-103.06%) | -3.00(-3.40--2.62) |
| Saint Kitts and Nevis | 59.00%(29.13%-96.41%) | -0.84(-1.14--0.53) | 47.67%(20.49%-81.40%) | -0.86(-1.15--0.56) | 71.62%(33.46%-115.41%) | -1.11(-1.44--0.78) |
| Saint Lucia | 111.76%(72.91%-155.98%) | -0.78(-1.08--0.47) | 107.84%(71.44%-150.84%) | -0.87(-1.18--0.55) | 111.28%(71.80%-157.11%) | -0.75(-1.06--0.44) |
| Saint Vincent and the Grenadines | 104.05%(66.96%-147.06%) | 0.21(-0.12-0.53) | 100.16%(64.23%-141.60%) | 0.17(-0.17-0.51) | 109.24%(70.45%-155.02%) | 0.22(-0.08-0.53) |
| Samoa | 32.93%(-0.15%-80.06%) | -1.00(-1.09--0.93) | 30.76%(-1.59%-74.52%) | -1.06(-1.14--0.98) | 30.68%(-4.86%-80.58%) | -1.08(-1.16--1.00) |
| San Marino | 88.03%(36.17%-154.44%) | 0.15(0.03-0.27) | 81.59%(16.50%-163.57%) | -0.12(-0.23-0.00) | 65.14%(0.93%-151.54%) | -0.20(-0.32--0.09) |
| Sao Tome and Principe | 171.27%(80.56%-263.38%) | 2.20(2.07-2.33) | 167.76%(78.83%-255.05%) | 2.22(2.09-2.36) | 178.88%(84.56%-284.37%) | 2.07(1.96-2.18) |
| Saudi Arabia | 247.29%(143.87%-435.57%) | 0.02(-0.25-0.29) | 172.79%(86.32%-330.26%) | -0.81(-1.09--0.52) | 212.76%(110.23%-402.38%) | -0.83(-1.07--0.59) |
| Senegal | 192.81%(96.15%-309.18%) | 1.41(1.09-1.73) | 193.24%(95.18%-307.89%) | 1.42(1.11-1.74) | 185.46%(89.33%-308.63%) | 1.29(0.96-1.62) |
| Serbia | 34.58%(2.84%-77.76%) | 0.17(-0.01-0.35) | 36.27%(4.50%-78.50%) | 0.12(-0.05-0.30) | 21.01%(-8.36%-61.96%) | 0.01(-0.17-0.20) |
| Seychelles | 103.20%(67.07%-150.08%) | -0.21(-0.36--0.06) | 92.74%(58.84%-138.56%) | -0.30(-0.45--0.15) | 101.84%(63.51%-148.45%) | -0.46(-0.60--0.33) |
| Sierra Leone | 143.46%(63.70%-234.22%) | 1.49(1.25-1.73) | 139.21%(60.78%-227.95%) | 1.48(1.24-1.73) | 151.10%(66.32%-255.70%) | 1.45(1.20-1.69) |
| Singapore | 63.41%(26.58%-109.65%) | -2.93(-3.22--2.63) | 20.87%(4.78%-40.36%) | -4.08(-4.41--3.75) | 9.51%(-5.56%-27.15%) | -4.27(-4.56--3.97) |
| Slovakia | 39.38%(0.64%-85.76%) | -0.47(-0.66--0.28) | 35.17%(-1.38%-80.57%) | -0.56(-0.74--0.38) | 26.55%(-8.66%-68.97%) | -0.78(-0.99--0.57) |
| Slovenia | 25.78%(-13.48%-81.69%) | -1.27(-1.42--1.13) | 15.90%(-20.05%-65.99%) | -1.66(-1.82--1.50) | 0.70%(-31.37%-45.02%) | -1.89(-2.06--1.72) |
| Solomon Islands | 117.34%(54.63%-205.07%) | -0.20(-0.32--0.07) | 115.14%(54.97%-201.95%) | -0.20(-0.32--0.07) | 119.73%(53.86%-210.81%) | -0.24(-0.37--0.12) |
| Somalia | 91.41%(30.75%-184.64%) | -1.00(-1.06--0.92) | 95.80%(36.33%-187.86%) | -0.92(-0.99--0.85) | 89.91%(31.28%-185.99%) | -1.03(-1.11--0.95) |
| South Africa | 52.50%(25.71%-125.22%) | -1.73(-2.39--1.08) | 56.15%(28.96%-124.22%) | -1.62(-2.27--0.96) | 37.79%(15.22%-107.86%) | -2.11(-2.77--1.43) |
| South Sudan | 19.49%(-15.08%-76.56%) | -1.21(-1.28--1.15) | 18.24%(-16.96%-74.90%) | -1.20(-1.27--1.14) | 21.40%(-17.65%-83.24%) | -1.32(-1.39--1.26) |
| Spain | 25.22%(-2.94%-60.53%) | -1.21(-1.28--1.14) | 15.29%(4.59%-27.40%) | -1.60(-1.70--1.50) | 0.19%(-9.47%-11.12%) | -1.96(-2.07--1.86) |
| Sri Lanka | 131.80%(58.07%-221.93%) | 0.45(0.05-0.86) | 117.79%(50.28%-199.49%) | 0.22(-0.20-0.65) | 96.06%(33.90%-173.37%) | -0.02(-0.46-0.41) |
| Sudan | 122.40%(55.69%-199.76%) | 0.40(0.28-0.51) | 120.20%(61.35%-193.92%) | 0.40(0.28-0.52) | 113.59%(51.32%-197.48%) | 0.17(0.05-0.28) |
| Suriname | 138.71%(88.77%-199.41%) | 0.03(-0.27-0.33) | 134.09%(84.81%-192.89%) | -0.03(-0.34-0.27) | 135.72%(86.06%-198.37%) | 0.00(-0.31-0.30) |
| Sweden | 54.33%(29.77%-81.30%) | 0.30(0.17-0.43) | 47.20%(36.09%-59.23%) | 0.11(0.00-0.22) | 40.35%(29.63%-51.95%) | 0.05(-0.09-0.19) |
| Switzerland | 34.13%(3.77%-73.89%) | -0.60(-0.70--0.48) | 22.07%(9.74%-36.55%) | -0.95(-1.08--0.81) | 7.83%(-3.69%-20.53%) | -1.35(-1.48--1.22) |
| Syrian Arab Republic | 140.34%(65.74%-256.82%) | -0.12(-0.33-0.10) | 133.86%(63.48%-247.25%) | -0.18(-0.39-0.03) | 121.84%(50.47%-236.56%) | -0.41(-0.64--0.18) |
| Taiwan  (Province of China) | 339.69%(232.08%-486.98%) | 2.68(2.40-2.94) | 269.76%(182.31%-393.65%) | 1.85(1.64-2.05) | 290.69%(193.46%-424.27%) | 2.56(2.29-2.84) |
| Tajikistan | -1.18%(-28.87%-111.64%) | -1.69(-2.16--1.21) | -2.75%(-30.46%-112.87%) | -1.60(-2.07--1.12) | 2.63%(-27.08%-115.76%) | -1.99(-2.46--1.52) |
| Thailand | 129.10%(63.10%-219.72%) | -1.38(-1.66--1.10) | 117.59%(55.43%-203.58%) | -1.69(-1.97--1.39) | 101.87%(42.33%-184.33%) | -1.55(-1.85--1.25) |
| Timor-Leste | 150.63%(73.49%-269.52%) | -0.39(-0.68--0.09) | 159.65%(80.69%-278.18%) | -0.35(-0.63--0.07) | 114.87%(45.02%-222.66%) | -0.61(-0.93--0.29) |
| Togo | 289.20%(165.32%-433.68%) | 1.30(1.14-1.46) | 285.89%(163.90%-426.07%) | 1.29(1.13-1.46) | 292.84%(163.60%-439.62%) | 1.28(1.12-1.43) |
| Tokelau | -16.83%(-36.15%-6.94%) | -0.62(-0.65--0.59) | -18.47%(-36.83%-3.83%) | -0.65(-0.68--0.62) | -18.21%(-39.01%-7.84%) | -0.82(-0.85--0.77) |
| Tonga | 47.64%(8.77%-98.47%) | -0.24(-0.58-0.10) | 50.15%(10.91%-99.46%) | -0.28(-0.62-0.06) | 39.51%(0.90%-90.80%) | -0.25(-0.59-0.08) |
| Trinidad and Tobago | 45.08%(4.37%-92.60%) | -2.04(-2.41--1.66) | 44.06%(4.12%-90.17%) | -2.11(-2.49--1.73) | 40.56%(-0.62%-89.37%) | -2.02(-2.38--1.66) |
| Tunisia | 174.77%(85.30%-285.94%) | 0.17(0.11-0.23) | 165.45%(79.38%-271.97%) | 0.01(-0.04-0.06) | 150.78%(67.82%-254.58%) | -0.07(-0.13-0.00) |
| Turkey | 75.71%(22.54%-153.77%) | -1.06(-1.29--0.83) | 69.30%(18.80%-142.46%) | -1.22(-1.43--1.02) | 47.10%(1.82%-120.19%) | -1.64(-1.87--1.41) |
| Turkmenistan | -38.91%(-52.10%--23.32%) | -4.46(-5.23--3.67) | -39.50%(-52.46%--24.41%) | -4.47(-5.24--3.69) | -37.97%(-51.70%--22.10%) | -4.47(-5.25--3.68) |
| Tuvalu | 18.12%(-14.43%-66.26%) | -0.95(-1.03--0.87) | 18.97%(-13.37%-66.47%) | -0.96(-1.03--0.88) | 12.54%(-19.95%-61.32%) | -1.04(-1.12--0.96) |
| Uganda | 169.71%(101.70%-265.39%) | 0.66(0.47-0.87) | 164.56%(99.37%-254.06%) | 0.65(0.48-0.84) | 173.77%(101.06%-277.43%) | 0.57(0.36-0.79) |
| Ukraine | -13.50%(-31.72%-8.06%) | -1.32(-1.76--0.88) | -16.89%(-34.34%-2.36%) | -1.53(-1.96--1.09) | -15.40%(-34.33%-5.47%) | -1.40(-1.89--0.91) |
| United Arab Emirates | 1088.98%(611.85%-1831.50%) | 0.26(-0.13-0.65) | 1044.14%(590.22%-1756.95%) | 0.20(-0.21-0.61) | 1128.50%(618.65%-1917.37%) | 0.21(-0.10-0.53) |
| United Kingdom | 58.37%(31.92%-90.08%) | 0.26(0.09-0.43) | 51.98%(45.93%-57.88%) | 0.02(-0.14-0.18) | 39.67%(35.10%-44.47%) | -0.15(-0.33-0.02) |
| United Republic of Tanzania | 79.44%(36.03%-138.38%) | -0.91(-0.98--0.84) | 79.22%(37.72%-136.48%) | -0.92(-0.98--0.84) | 78.09%(32.10%-141.84%) | -0.98(-1.05--0.91) |
| United States of America | 93.21%(63.16%-126.50%) | 0.14(0.00-0.28) | 89.82%(82.50%-97.66%) | 0.09(-0.04-0.21) | 76.57%(69.94%-83.26%) | -0.16(-0.27--0.05) |
| United States Virgin Islands | 166.48%(101.89%-243.76%) | 1.25(0.92-1.58) | 168.24%(105.05%-245.09%) | 1.15(0.83-1.47) | 138.16%(76.18%-210.67%) | 1.23(0.88-1.57) |
| Uruguay | -9.19%(-30.02%-16.23%) | -1.65(-1.79--1.51) | -8.83%(-18.79%-2.64%) | -1.75(-1.90--1.61) | -17.44%(-26.66%--7.57%) | -1.87(-2.02--1.74) |
| Uzbekistan | -27.32%(-39.67%--13.20%) | -4.05(-4.52--3.57) | -29.59%(-41.46%--16.37%) | -3.98(-4.46--3.50) | -23.21%(-36.37%--8.05%) | -4.26(-4.73--3.78) |
| Vanuatu | 164.39%(90.53%-267.62%) | -0.22(-0.36--0.08) | 164.23%(92.18%-264.66%) | -0.22(-0.34--0.09) | 160.11%(87.85%-270.07%) | -0.25(-0.40--0.10) |
| Venezuela  (Bolivarian Republic of) | 148.00%(79.81%-227.63%) | -0.87(-1.04--0.68) | 142.81%(78.24%-220.37%) | -1.00(-1.19--0.82) | 138.00%(70.07%-219.32%) | -0.88(-1.06--0.69) |
| Viet Nam | 210.59%(117.41%-336.31%) | 1.25(1.08-1.41) | 190.26%(105.35%-306.48%) | 1.03(0.87-1.18) | 207.01%(108.46%-343.02%) | 1.16(0.97-1.35) |
| Yemen | 160.54%(83.92%-285.17%) | -0.11(-0.14--0.08) | 161.54%(86.07%-283.11%) | -0.10(-0.12--0.07) | 153.34%(74.56%-285.27%) | -0.26(-0.30--0.22) |
| Zambia | 88.75%(38.73%-164.98%) | -1.18(-1.44--0.92) | 86.85%(38.35%-160.76%) | -1.18(-1.44--0.93) | 90.41%(36.65%-172.65%) | -1.24(-1.53--0.96) |
| Zimbabwe | 80.92%(36.21%-129.97%) | 0.06(-0.22-0.35) | 78.81%(34.47%-126.21%) | 0.06(-0.18-0.29) | 89.37%(39.04%-145.55%) | 0.22(-0.04-0.49) |

Abbreviations: ASIR, age-standardized incidence rate; ASDR, age-standardized death rate; CI, confidential interval; DALY, disability-adjusted life-years; EAPC, estimated annual percentage change; UI, uncertain interval.
