# Supplementary material for: Burden of esophageal cancer and its attributable risk factors in 204 countries and territories from 1990 to 2019
Source: Front Public Health. 2022 Sep 6;10:952087. doi: 10.3389/fpubh.2022.952087 (PMC9485842; doi:10.3389/fpubh.2022.952087)
Supplement: Supplementary file 3 [file Table_3.docx]

**Supplementary Table 3. Contributions of different risk factors to esophageal cancer deaths globally and in five sociodemographic index (SDI) quintiles in 2019.**

|  | **Smoking** | **Alcohol use** | **High body mass index** | **Diet low in fruits** | **Diet low in vegetables** |
| --- | --- | --- | --- | --- | --- |
| **Global** | 40.6%(36.8%-44.3%) | 22.6%(17.2%-27.9%) | 17.9%(5.7%-35.0%) | 10.3%(3.1%-22.2%) | 3.5%(0.5%-6.9%) |
| **High SDI** | 42.7%(38.4%-47.1%) | 31.6%(24.2%-38.4%) | 26.2%(8.6%-45.8%) | 8.8%(1.8%-22.0%) | 4.8%(0.3%-10.2%) |
| **High-middle SDI** | 45.8%(41.5%-50.4%) | 25.9%(19.8%-31.9%) | 18.7%(5.6%-35.9%) | 7.6%(1.7%-19.0%) | 1.6%(0.3%-3.3%) |
| **Middle SDI** | 41.8%(37.8%-46.0%) | 20.6%(15.7%-25.6%) | 15.6%(4.8%-31.8%) | 9.4%(2.4%-21.7%) | 2.0%(0.3%-4.1%) |
| **Low-middle SDI** | 30.8%(27.0%-35.3%) | 13.1%(9.7%-16.8%) | 14.7%(4.7%-29.1%) | 18.7%(8.1%-29.7%) | 7.3%(1.0%-14.3%) |
| **Low SDI** | 20.6%(16.8%-24.8%) | 12.7%(9.1%-16.7%) | 13.8%(4.1%-27.3%) | 16.1%(6.2%-27.3%) | 10.2%(1.8%-19.2%) |
