# Supplementary material for: Burden of esophageal cancer and its attributable risk factors in 204 countries and territories from 1990 to 2019
Source: Front Public Health. 2022 Sep 6;10:952087. doi: 10.3389/fpubh.2022.952087 (PMC9485842; doi:10.3389/fpubh.2022.952087)
Supplement: Supplementary file 8 [file Data_Sheet_1.docx]

**Text S1. Supplemental Methods**
**1.Estimation of deaths**

**Input data**

The cause of death (COD) database contains multiple sources of cancer mortality data, including vital registration, verbal autopsy, and cancer registry data. The cancer registry mortality estimates that are uploaded into the COD database stem from cancer registry incidence data that have been transformed to mortality estimates through the use of mortality-to-incidence ratios (MIR).

**Modelling strategy**


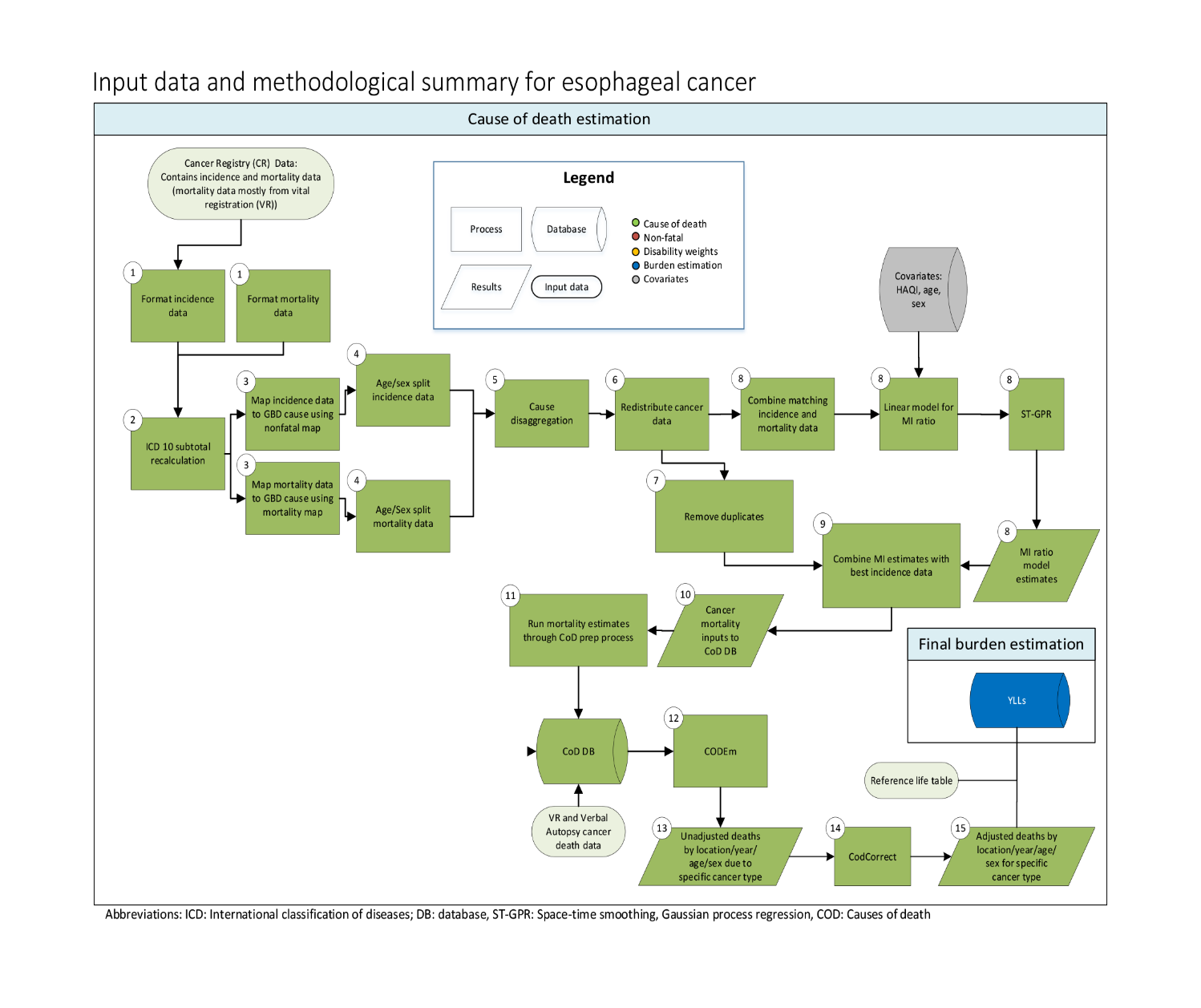


**2. Non-fatal Estimation**

**Case definition**

All cancers coded as 150-150.9, 211.0, 230.1 in the 9th revision of the International Classification of Diseases and Injuries (ICD-9) or C15-C15.9 and D00.1, D13.0 in the ICD-10 are recognized as esophageal cancer. Prevalence for esophageal cancer is estimated for a maximum of 10 years after incidence.

Total prevalence for esophageal cancer is split into four sequelae:1. diagnosis and primary therapy; 2. controlled phase; 3. metastatic phase; and 4. terminal phase. The diagnosis and primary therapy phase is defined as the time from the onset of symptoms to the end of treatment. The controlled phase is defined as the time between finishing primary treatment and the earliest of either: cure (defined as recurrence-and progression-free survival after 10 years); death from another cause; or progression to the metastatic phase. The metastatic phase is defined as the time period of intensive treatment for metastatic disease, as determined for each cancer by SEER (Surveillance, Epidemiology, and End Results Program) averages. The terminal phase is defined as the one-month period prior to death. Each of these four sequelae has a separate disability weight.

**Duration of four prevalence sequelae**

|  | Diagnosis and primary therapy(months) | Controlled phase(months) | Metastatic phase(months) | Terminal phase(months) |
| --- | --- | --- | --- | --- |
| Esophageal cancer | 5 | Calculated based on remainder of time after attributing other sequelae. | 4.6(SEER Summary Stage 1997  (Distant site/node involved)1995-2000) | 1 |

**Disability weights**

| Health state | Lay description | Estimate |
| --- | --- | --- |
| Diagnosis and primary therapy | This person has pain, nausea, fatigue, weight loss and high anxiety. | 0.288(0.193-0.399) |
| Controlled phase | This person has a chronic disease that requires medication every day and causes some worry but minimal interference with daily activities. | 0.049 (0.031–0.072) |
| Metastatic phase | This person has severe pain, extreme fatigue, weight loss and high anxiety | 0.451 (0.307–0.600) |
| Terminal phase | This person has lost a lot of weight and regularly uses strong medication to avoid constant pain. The person has no appetite, feels nauseous, and needs to spend most of the day in bed. | 0.540 (0.377–0.687) |

**Modelling strategy**


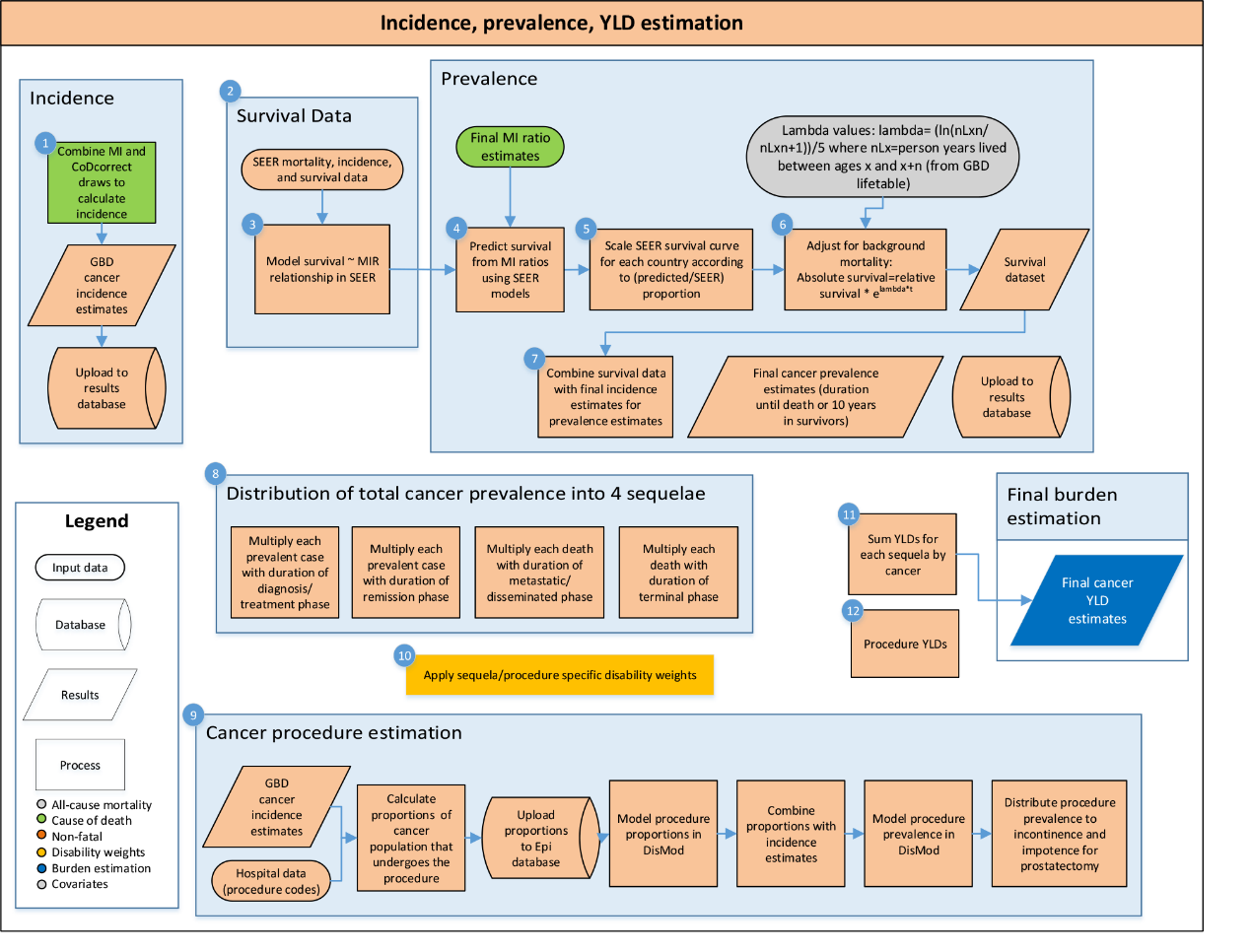


Risk factors

Relative risk and exposure estimate for risk factors are extracted from following data sources: randomized controlled trials, cohort studies, household surveys, census data, and other data sources. The proportions of esophageal cancer related deaths attributable to potentially modifiable risk factors are estimated by the comparative risk assessment conceptual framework. The counterfactual scenario of theoretical minimum is used to model the population attributable fraction, which reflects the proportional reduction in age-standardized deaths that would occur if the exposure to a risk factor is reduced to the theoretical minimum exposure level. The theoretical minimum risk exposure level is defined as the level of risk exposure that minimizes risk at the population level or the level of risk that captures the maximum attributable burden.

**Table.** Definition and theoretical minimum risk exposure level for 5 risk factors of esophageal cancer

| **Risk factors** | **Definition** | **Theoretical minimum risk exposure level** |
| --- | --- | --- |
| Smoking | Prevalence of current use of any smoked tobacco product and prevalence of former use of any smoked tobacco product; among current smokers, cigarette equivalents smoked per smoker per day and cumulative pack-years of exposure; among former smokers, number of years since quitting | All individuals are lifelong non smokers |
| Alcohol use | Grams of alcohol consumed by current drinkers, per day, over a12-month period | The exposure that minimizes the risk of suffering burden from any given cause related to alcohol |
| High body mass index | Defined as BMI greater than 20 to 25 kg/m2 for adults (ages 20+). | 20 to 25 kg/m2 |
| Diet low in vegetables | Average daily consumption (in grams per day) of less than280-320gramsof vegetables, including fresh, frozen, cooked, canned, or dried vegetables and excluding legumes and salted or pickled vegetables, juices, nuts and seeds, and starchy vegetables such as potatoes or corn | 290-430gr/day |
| Diet low in fruits | Averagedaily consumption (in grams per day) of less than310-340gramsof fruit including fresh, frozen, cooked, canned, or dried fruit, excluding fruit juices and salted or pickled fruits | 200-300gr/day |

**References**

1. Global burden of 369 diseases and injuries in 204 countries and territories, 1990-2019: a systematic analysis for the Global Burden of Disease Study 2019. Lancet (London, England) 2020, 396(10258):1204-1222.

2. Global burden of 87 risk factors in 204 countries and territories, 1990-2019: a systematic analysis for the Global Burden of Disease Study 2019. Lancet (London, England) 2020, 396(10258):1223-1249.

3. Global age-sex-specific fertility, mortality, healthy life expectancy (HALE), and population estimates in 204 countries and territories, 1950-2019: a comprehensive demographic analysis for the Global Burden of Disease Study 2019. Lancet (London, England) 2020, 396(10258):1160-1203.
